# Agreement and Screening Performance of an App-Based Cranial Shape Assessment Tool Compared With a 3D Scanner in Municipal Child Health Checkups in Japan: Instrument Validation Study

**DOI:** 10.2196/105473

**Published:** 2026-10-13

**Authors:** Yui Honda, Mai Takeshita, Hiroki Kajita, Yoriko Kotoku, Kayo Ueda, Shigeharu Hosono, Hiromi Eto

**Affiliations:** 1 Department of Reproductive Health, Graduate School of Biomedical Sciences, Nagasaki University, Nagasaki, Japan; 2 Department of Implementation Science in Public Health, Kyoto University School of Public Health, Kyoto University, Yoshida Konoe-cho, Sakyo-ku, Kyoto, 6068501, Japan, 81 9069022390; 3 Department of Plastic and Reconstructive Surgery, Keio University School of Medicine, Keio University, Tokyo, Japan; 4 Baby’s Head Reshaping Clinic, Fukuoka, Japan; 5 Department of Nursing Sciences, Graduate School of Biomedical Sciences, Nagasaki University, Nagasaki, Japan; 6 Department of Reproductive Health, Institute of Biomedical Sciences, Nagasaki University, Nagasaki, Japan; 7 Department of Pediatrics, Nerima Hikarigaoka Hospital, Tokyo, Japan; 8 Jichi Medical University, Tochigi, Japan

**Keywords:** plagiocephaly, infants, mobile apps, 3D imaging, cranial vault asymmetry index, cephalic index, screening performance, reproducibility, sensitivity, specificity

## Abstract

**Background:**

Positional cranial deformities are common in infancy. Smartphone-based apps are increasingly used to assess infant head shape, but evidence comparing these tools with 3D scanners across age groups in community-based settings remains limited.

**Objective:**

This study evaluated age-stratified agreement and screening performance of a mobile app for estimating Cranial Vault Asymmetry Index (CVAI) and Cephalic Index, compared with a 3D scanner, when used by a trained examiner during routine municipal health checkups in Japan.

**Methods:**

We conducted a cross-sectional study in Goto City, Japan, between May 2023 and March 2025. Independent, age-specific cohorts of children attending municipal health checkups at 4, 18, and 42 months were included. Paired cranial measurements were obtained by a trained examiner using a VECTRA H2 3D scanner (Canfield Scientific Inc) and the mobile app. Agreement was assessed using mean differences, 95% limits of agreement (LoA), and intraclass correlation coefficients (ICCs), with device-stratified sensitivity analyses. Screening performance was evaluated using sensitivity and specificity based on prespecified clinical thresholds. Receiver operating characteristic (ROC) analyses were secondary exploratory analyses. The primary outcome was app-based CVAI for detecting scanner-defined moderate-or-worse plagiocephaly (CVAI≥7%). Secondary outcomes were app-based Cephalic Index for detecting brachycephaly (Cephalic Index>94%) and dolichocephaly (Cephalic Index<79%).

**Results:**

The final analytic samples comprised 262 children at 4 months, 171 at 18 months, and 216 at 42 months. Agreement for continuous CVAI was highest at 4 months (mean difference 0.14, 95% LoA −4.18 to 4.46; ICC 0.76, 95% CI 0.71‐0.81) and lower at 18 months (ICC 0.42, 95% CI 0.29‐0.54) and 42 months (ICC 0.49, 95% CI 0.38‐0.59). For Cephalic Index, the app consistently overestimated values relative to the scanner, with mean differences of 3.83 SD 3.42, 3.24 SD 3.07, and 2.61 SD 3.33 points at 4, 18, and 42 months, respectively. For detecting scanner-defined moderate-or-worse plagiocephaly, app sensitivity and specificity were 84.8% and 88.0% at 4 months, 50.0% and 88.4% at 18 months, and 53.3% and 89.6% at 42 months, respectively. Device-stratified analyses showed some variation in measurement agreement and screening performance across devices.

**Conclusions:**

Under municipal health checkup conditions with a trained examiner, app-based CVAI showed the strongest performance at 4 months and may be useful for early screening. However, performance was weaker at 18 and 42 months. App-based Cephalic Index was systematically higher than scanner-based Cephalic Index, potentially increasing false-positive classifications for brachycephaly when identical thresholds are applied. Performance varied somewhat between devices, although device effects could not be separated from study period and accumulated examiner experience. Positive findings, particularly at later ages or for Cephalic Index–based measures, should be interpreted in the context of the child’s age and broader clinical findings, with further evaluation or referral considered only when clinically indicated.

## Introduction

Positional cranial deformities, including plagiocephaly, brachycephaly, and dolichocephaly, are common conditions observed during infancy. Although some cases may resolve spontaneously over time, moderate-to-severe deformities may warrant early evaluation and, when appropriate, timely consideration of cranial orthotic therapy within a critical age window during which treatment effectiveness is expected to be greater [1-3]. In Japan, parental awareness and concern regarding infant head deformities have increased, and the number of head-shape consultations has risen in recent years [4,5]. In this context, the availability of accessible and reliable tools to support timely assessment and appropriate referral decisions has become increasingly important.

Cranial shape is commonly quantified using standardized indices. For plagiocephaly, the Cranial Vault Asymmetry Index (CVAI) is widely used to quantify cranial asymmetry, whereas the Cephalic Index is used to describe head width-to-length proportions relevant to the classification of brachycephaly and dolichocephaly. In Japan, threshold-based classification systems derived from these indices are routinely applied in clinical and community settings, including the identification of moderate-or-worse deformities that may prompt specialist referral or closer clinical follow-up [6,7]. However, the application of index-based thresholds depends on the availability of measurements that demonstrate sufficient reliability and consistency under real-world conditions, particularly in nonspecialist, community-based settings.

Smartphone- and tablet-based apps that estimate cranial indices from photographs are increasingly available and widely accessible. Such apps have the potential to serve as practical screening tools; however, their accuracy may be influenced by real-world image capture conditions and may vary across different age groups [8-10]. 3D scanners are generally regarded as the reference standard for cranial shape assessment, as they reconstruct head morphology in three dimensions and derive indices in a standardized and reproducible manner [7]. Nevertheless, empirical evidence directly comparing app-based measurements with 3D scanner-derived indices in real-world populations of infants and young children—particularly across different ages and within community-based settings—remains limited.

In Japan, municipal child health checkups provide a near-universal platform for early childhood health assessment and monitoring. The 18-month checkup and the checkup for children aged 3 years to younger 4 years are mandated by law, and a 3‐ to 5-month infant checkup is also widely implemented across municipalities. Recent national data indicate attendance rates of approximately 96% for the 3‐ to 5-month, 18-month, and 3-year checkups [11]. Accordingly, these settings provide opportunities not only for early identification of cranial deformities during infancy but also for follow-up, parental reassurance, and guidance regarding residual head shape asymmetry in early childhood.

Therefore, this study aimed to evaluate whether this mobile app could serve as a practical screening aid under municipal health checkup conditions by (1) quantifying age-stratified agreement in continuous CVAI and Cephalic Index measurements between a 3D scanner and the mobile app and (2) assessing its screening performance relative to 3D-scanner–based reference classifications for deformational plagiocephaly, brachycephaly, and dolichocephaly.

## Methods

### Study Design and Setting

This cross-sectional study included infants and young children attending municipal health checkups in Goto City, Nagasaki Prefecture, Japan. The 4-, 18-, and 42-month assessments were treated as independent cross-sectional samples, with no child contributing to more than one age group. A formal a priori sample size calculation for diagnostic accuracy estimates was not conducted because all eligible participants attending the age-specific checkups during the study period were targeted for enrollment.

All measurements were obtained within routine municipal health checkup workflows under standardized conditions. The 4-month checkups were conducted at the obstetrics and gynecology outpatient clinic of Goto Central Hospital, whereas the 18- and 42-month checkups were conducted at the Fukue Comprehensive Welfare and Health Center. Reporting was guided by the STROBE (Strengthening the Reporting of Observational Studies in Epidemiology) statement for observational studies and the STARD (Standards for Reporting Diagnostic Accuracy Studies) 2015 guideline [12,13]. Completed checklists are provided in Checklist 1 (STROBE checklist) and Checklist 2 (STARD checklist).

### Participants

Eligible participants were infants and young children, along with their parents or guardians, who attended the 4-, 18-, or 42-month municipal health checkups organized by Goto City between May 2023 and March 2025. Children were excluded if they had a diagnosed congenital cranial disorder or an obvious structural abnormality (eg, craniosynostosis).

These age-specific municipal health checkups have high population coverage, with nearly all eligible children in the target age groups attending. After routine clinical examination and anthropometric measurements, a research nurse or midwife approached parents or guardians to invite participation and obtained written informed consent prior to study-specific measurements.

### Data Collection Procedures

Parents or guardians completed a self-administered questionnaire on obstetric history, the child’s medical history, and daily positioning and care practices. Additional variables collected for descriptive and comparative purposes included the child’s sex, mode of delivery, gestational age at birth, birth weight, and anthropometric measurements at the time of the health checkup (ie, weight, length or height [as appropriate], and head circumference), as well as the presence of muscular torticollis or limitation of hip abduction as assessed by physicians and documented in health checkup records. Information on birth-related and anthropometric variables was extracted from the Maternal and Child Health Handbook and health checkup records. The handbook is a nationally standardized, home-based record system used in Japan to document pregnancy, birth, and child health information, and is routinely referenced during municipal health checkups [14-16].

Cranial measurements were performed after fitting a standardized measurement cap and were obtained using both the mobile app and the 3D scanner during the same visit. All measurements were performed by a trained examiner following a predefined protocol. To minimize burden on children and parents and disruption to routine health checkup procedures, measurements were performed as efficiently as possible and were omitted if the child was excessively distressed or uncooperative.

### Reference Standard: 3D Scanner

3D scanners are widely used as the clinical reference method for quantitative cranial shape assessment; however, they do not represent a pathological gold standard independent of measurement. We used the VECTRA H2 3D scanner (Canfield Scientific Inc), which has been used clinically for cranial shape assessment [17,18]. Each child wore a dedicated grid-pattern measurement cap to reduce interference from hair and facilitate 3D surface reconstruction. Children aged 4 and 18 months were imaged while seated on their caregiver’s lap, whereas children aged 42 months were seated in a chair. Imaging was conducted by a single trained examiner with assistance from one staff member. All scans were performed throughout the study period by the same examiner, who received prestudy training using a head model.

Cranial images were captured from 7 directions for construction of a 3D cranial model. Subsequent processing and quantitative analysis of the 3D scanner data were performed by Japan Medical Company (JPMC) technical staff according to previously reported methods [7,19,20]. 3D image reconstruction was performed primarily using dedicated software, with manual refinement by technical staff as needed. Subsequent procedures included definition of measurement planes and landmarks, measurement of cranial dimensions, calculation of CVAI and Cephalic Index, and severity classification. A reference plane (0th slice) was defined by connecting the lowest point of the nasal root (ie, the sellion) and the bilateral tragion points, after which the cranium was divided into 10 equal horizontal slices, with the vertex corresponding to the 10th slice. The third slice from the reference plane was used for quantitative analysis. Cranial asymmetry (CA, mm), CVAI using the shorter diagonal as the denominator (CVAI(S), where S indicates the shorter diagonal; %), and Cephalic Index (%) were derived at this slice. CA was defined as the absolute difference between the lengths of the two 30° diagonal lines across the midline. CVAI(S) was calculated as the difference in diagonal lengths divided by the shorter diagonal, expressed as a percentage. Hereafter, CVAI(S) is referred to as CVAI. Cephalic Index was calculated as biparietal width divided by anteroposterior length, expressed as a percentage. Scanner-derived CVAI and Cephalic Index were used as the reference-standard measures for agreement and screening performance analyses.

### Index Test: Mobile App

The index test was the “Akachan no Atama no Katachi SOKUTEI (Measurement of Baby’s Head Shape)” mobile app, developed by JPMC as a nonmedical, smartphone- or tablet-based tool for assessing infant cranial shape for screening purposes. The app was installed on dedicated tablets. Images captured from May 22, 2023, to June 24, 2024, were obtained using an Apple iPad (sixth generation; iOS 17.5.1), whereas images captured from June 25, 2024, to March 31, 2025, were obtained using a Lenovo Tab M10 Plus (third generation; TB125FU; Android 13). The device change was not preplanned and occurred because the in-app camera function could no longer be launched on the original iPad. The Lenovo tablet was selected as the next available device confirmed to be compatible with the app. For the main analyses, data from both device periods were pooled to evaluate overall performance across the municipal screening program; device-stratified analyses were conducted as sensitivity analyses.

Although the app is intended for use by parents or caregivers as well as health care professionals, all image capture, landmark identification, and contour tracing in this study were performed by a single trained examiner. App-based measurements were performed after 3D scanning by the same examiner using the same measurement cap and positioning, with minimal delay between measurements. The vertex image of the child’s head was captured using the tablet’s built-in camera. The examiner premarked the positions of both ears and the nose on the measurement cap and, after image capture, identified these landmarks on the two-dimensional image and manually traced the cranial contour according to the in-app instructions (Multimedia Appendix 1). The app outputs 2 indices: “Yugami-do” (degree of asymmetry) and “Tantou-do” (degree of brachycephaly). Based on developer information and previous validation work, these indices were operationally defined and analyzed as app-based CVAI and app-based Cephalic Index, respectively [8].

### Blinding and Test Interpretation

At the time of app-based image capture and contour tracing, the examiner did not have access to the final, processed quantitative results derived from the 3D scanner. Conversely, JPMC technical staff who processed and analyzed the 3D scanner data were blinded to the app-based measurement results. However, because the same examiner performed both procedures consecutively in a fixed order under a partially shared measurement context, the measurements were not fully independent and may have been subject to measurement bias.

### Definitions of Cranial Indices and Clinical Thresholds

Plagiocephaly severity was classified according to the CVAI severity criteria specified in the Japanese package insert for the Michigan-style cranial reshaping orthosis: normal (<5%), mild (5%-<7%), moderate (7%-<10%), severe (10%-<14%), and very severe (≥14%) [21].

For the primary screening accuracy analyses, moderate-or-worse plagiocephaly was defined as scanner-derived CVAI≥7%. This prespecified threshold represents clinically meaningful severity for which cranial orthotic (helmet) therapy may be considered [7].

Brachycephaly was defined as scanner-derived Cephalic Index>94% and dolichocephaly as Cephalic Index<79%, based on reference values for Japanese children and thresholds used in previous Japanese clinical studies [7,22]. These thresholds were selected to align with population-specific normative data and established clinical interpretations in Japan. Because age-specific differences in Cephalic Index have been reported among Japanese children [22], analyses were stratified by age at 4, 18, and 42 months.

### Statistical Analysis

All statistical analyses were performed using SPSS Statistics (version 29.0; IBM Corp). Agreement, screening performance, and receiver operating characteristic (ROC) analyses were conducted separately within each age group, using participants with paired measurements from both methods for the relevant index. Participant characteristics were summarized as counts and percentages for categorical variables and means and SDs for continuous variables. Continuous variables were reviewed for distributional plausibility to support the use of parametric summaries.

### Primary Analysis: Agreement (Continuous Indices)

Agreement between the 3D scanner and mobile app was assessed for CVAI and Cephalic Index within each age group. Differences were calculated as app−scanner, with the mean difference representing systematic bias. Positive values indicated higher measurements from the app relative to the 3D scanner.

Bland-Altman 95% limits of agreement (LoA) were computed as mean difference ±1.96×SD of the differences, and Bland-Altman plots were generated for visual inspection of agreement and potential trends across the measurement range. Systematic and proportional bias were assessed descriptively from the plots. Heteroscedasticity was assessed using Spearman rank correlations between the absolute method differences and measurement magnitude, defined as the mean of the 2 methods.

Intraclass correlation coefficients (ICCs) were estimated within each age group using a two-way mixed-effects model with absolute agreement for single measurements (ICC [1,3]) and reported with 95% CIs [23]. ICCs were interpreted according to conventional thresholds: <0.50, poor; 0.50‐0.75, moderate; 0.75‐0.90, good; >0.90, excellent agreement. ICCs were calculated from paired measurements only, using listwise deletion within each age group and index.

### Primary Analysis: Screening Performance (Clinically Anchored Thresholds)

For screening performance analyses, 3D scanner–based classifications were used as the reference standard (plagiocephaly: CVAI≥7%; brachycephaly: Cephalic Index>94%; dolichocephaly: Cephalic Index<79%). App-based results were dichotomized using the same thresholds. For each age group, 2×2 contingency tables were constructed to calculate scanner-defined prevalence, sensitivity, specificity, positive predictive value (PPV), and negative predictive value (NPV). For sensitivity, specificity, PPV, and NPV, 95% CIs were calculated using the Wilson score method.

### Secondary Exploratory ROC Analyses

As secondary exploratory analyses, ROC analyses evaluated continuous app-based indices against scanner-defined classifications within each age group. Areas under the curve (AUCs) with 95% CIs were estimated, and optimal cutoffs were identified by maximizing the Youden index (sensitivity + specificity − 1). Sensitivity-prioritized cutoffs were also derived.

For outcomes in which higher app-based values indicated a positive state (CVAI and brachycephaly), the sensitivity-prioritized cutoff was defined as the highest cutoff achieving sensitivity≥0.90 while maximizing specificity; for dolichocephaly, in which lower values indicated a positive state, it was defined as the lowest cutoff meeting the same criterion.

For plagiocephaly, we additionally performed ROC analyses using multiple scanner-defined severity thresholds as the positive definition (CVAI≥5%,≥7%,≥10%, and≥14%). We summarized the AUC, Youden-optimal cutoff values, and the corresponding sensitivity and specificity for each threshold. Age-stratified confusion matrices were also constructed using identical classification boundaries for both methods: 5×5 matrices for plagiocephaly (normal,<5%; mild, 5%-<7%; moderate, 7%-<10%; severe, 10%-<14%; very severe,≥14%) and 3×3 matrices for cephalic shape (dolichocephaly, Cephalic Index<79%; normal, 79%‐94%; brachycephaly, Cephalic Index>94%). For both, the 3D scanner classifications were represented as rows and app classifications as columns, and cell counts, row percentages, exact agreement rates, and directions of discordance (overclassification vs underclassification relative to the 3D scanner reference) were reported.

### Sensitivity Analyses: Device-Stratified Analyses

To assess potential variation associated with the tablet device, sensitivity analyses were stratified by the iPad and Lenovo device periods described above. Within each device period and each age group (4, 18, and 42 months), we repeated the primary agreement analyses (mean difference [app−scanner], Bland-Altman 95% LoA, and ICC [1,3] with 95% CIs) and the threshold-based screening performance analyses (sensitivity and specificity, with 95% CIs).

### Ethical Considerations

This study was conducted in accordance with the Declaration of Helsinki and the Japanese Ethical Guidelines for Life Science and Medical Research Involving Human Subjects. The protocol was approved by the Ethics Committee of the Graduate School of Biomedical Sciences, Nagasaki University (approval no. 23020901‐3) and prospectively registered in the University Hospital Medical Information Network (UMIN) Clinical Trials Registry (UMIN000052342). Written informed consent was obtained from all parents or legal guardians after they received a comprehensive explanation of the study aims, procedures, potential risks and benefits, and the voluntary nature of participation, including explicit assurance that refusal or withdrawal would not affect access to health services. When results indicated severe or very severe cranial asymmetry, parents or guardians were offered referral for further clinical assessment and provided with information on specialist services, with referral facilitated as appropriate. Low-dose computed tomography (CT) cranial imaging was considered when clinically indicated.

## Results

### Participant Characteristics

Figure 1 shows the flow of participants through the study. Of the consenting participants, 7 of 269 children at 4 months, 11 of 182 at 18 months, and 3 of 219 at 42 months were excluded because 3D scanner images could not be successfully reconstructed, resulting in final analytic samples of 262, 171, and 216 children, respectively. Agreement and screening performance analyses were performed using paired measurements from both the 3D scanner and the mobile app for each index. At 18 months, 1 participant had missing app-based Cephalic Index data and was therefore excluded from Cephalic Index−based analyses only, while remaining included in CVAI-based analyses.

**Figure 1.**
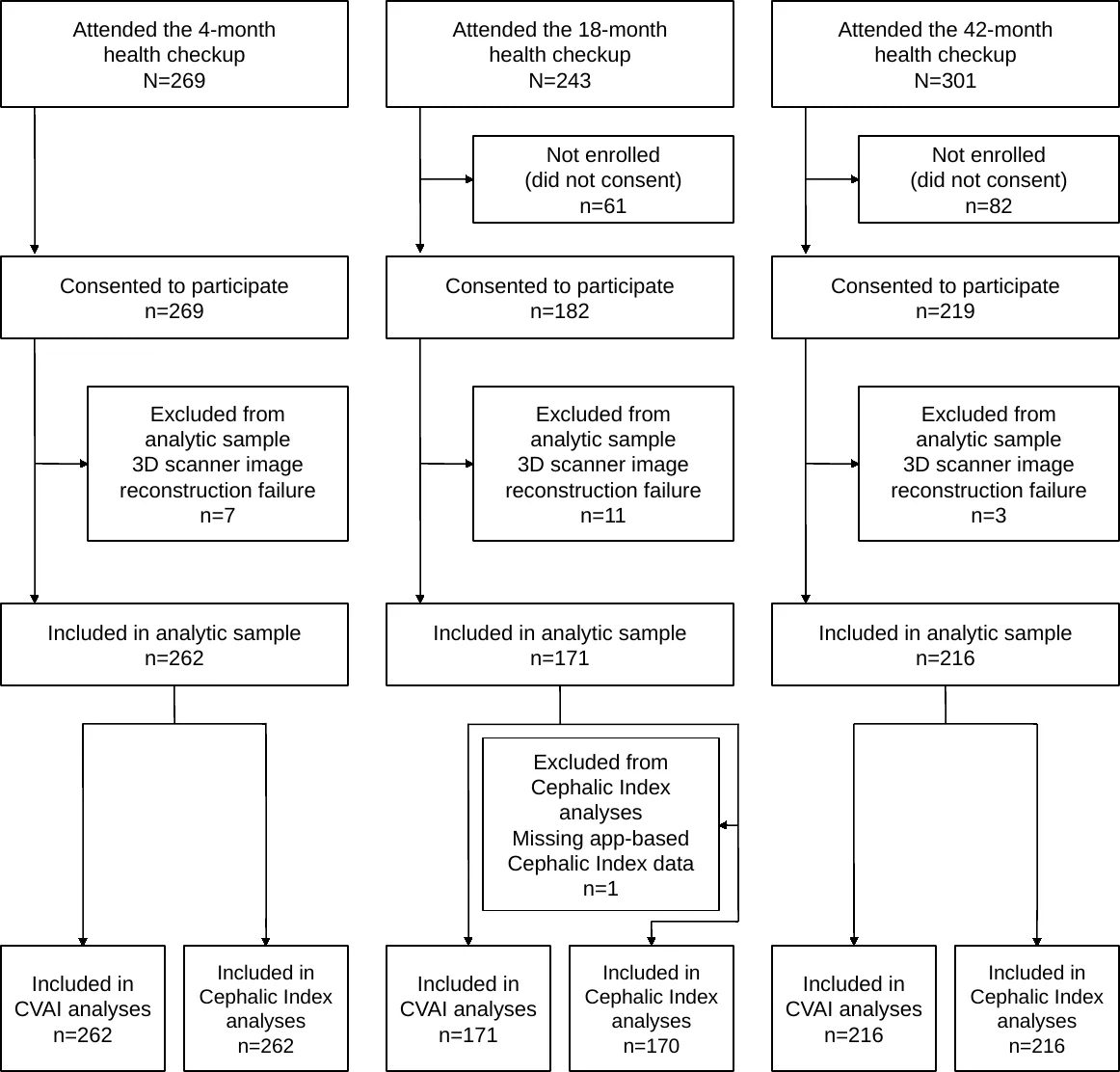
Participant flow diagram by age group. CVAI: Cranial Vault Asymmetry Index.

The characteristics of the participants at each assessment time point are shown in Table 1. Across all 3 assessments, the sex distribution was approximately balanced, with boys accounting for 49.6% (130/262) at 4 months, 45.0% (77/171) at 18 months, and 45.1% (97/215) at 42 months. The proportion of children born by cesarean section ranged from 13.5% (29/215) to 18.7% (32/171), indicating that the majority of participants were delivered vaginally. Mean gestational age at birth was consistent with term delivery at all time points (mean 39.1, SD 1.7, 38.9, SD 1.7, and 39.2, SD 1.7, weeks at 4, 18, and 42 months, respectively). Torticollis and limitation of hip abduction were rarely observed, with no more than 1 child affected at any assessment time point (0%-0.5%). Missing data for selected variables were minimal and are indicated in the table footnotes.

**Table 1.** Characteristics of participants at each assessment time point^a^.

Characteristics	4 months (n=262)^b^	18 months (n=171)^c^	42 months (n=216)^d^
Sex, n (%)			
Male	130 (49.6)	77 (45.0)	97 (45.1)
Female	132 (50.4)	94 (55.0)	118 (54.9)
Delivery			
Vaginal delivery, n (%)	218 (83.8)	139 (81.3)	186 (86.5)
Cesarean section, n (%)	42 (16.2)	32 (18.7)	29 (13.5)
Gestation, at birth, weeks, mean (SD)	39.1 (1.7)	38.9 (1.7)	39.2 (1.7)
Weight at assessment, kg, mean (SD)	6.9 (0.8)	10.4 (1.0)	14.4 (1.8)
Height at assessment, cm, mean (SD)	62.9 (2.6)	79.5 (2.7)	94.5 (5.5)
Head circumference, cm, mean (SD)	41.6 (1.3)	47.2 (1.3)	49.8 (1.5)
Torticollis, n (%)	0 (0)	0 (0)	1 (0.5)
Limitation of hip abduction, n (%)	1 (0.4)	0 (0)	1 (0.5)

a Percentages were calculated using the number of participants with nonmissing data for each variable.

b At 4 months, data on mode of delivery were missing for 2 participants.

c At 18 months, data on weight and height at assessment were missing for 1 participant each.

d At 42 months, data on sex, mode of delivery, torticollis, and limitation of hip abduction were missing for 1 participant each.

### Distribution of Cranial Indices by Age Group and Measurement Method

Figure 2 shows the age-stratified distributions of CVAI (%) obtained using the 3D scanner and mobile app. Across age groups, CVAI values were right-skewed and concentrated at lower levels, and the app tended to yield slightly higher values than the 3D scanner. For the 3D scanner, most CVAI values were distributed below the higher severity thresholds, with relatively few observations in the severe and very severe ranges.

**Figure 2.**
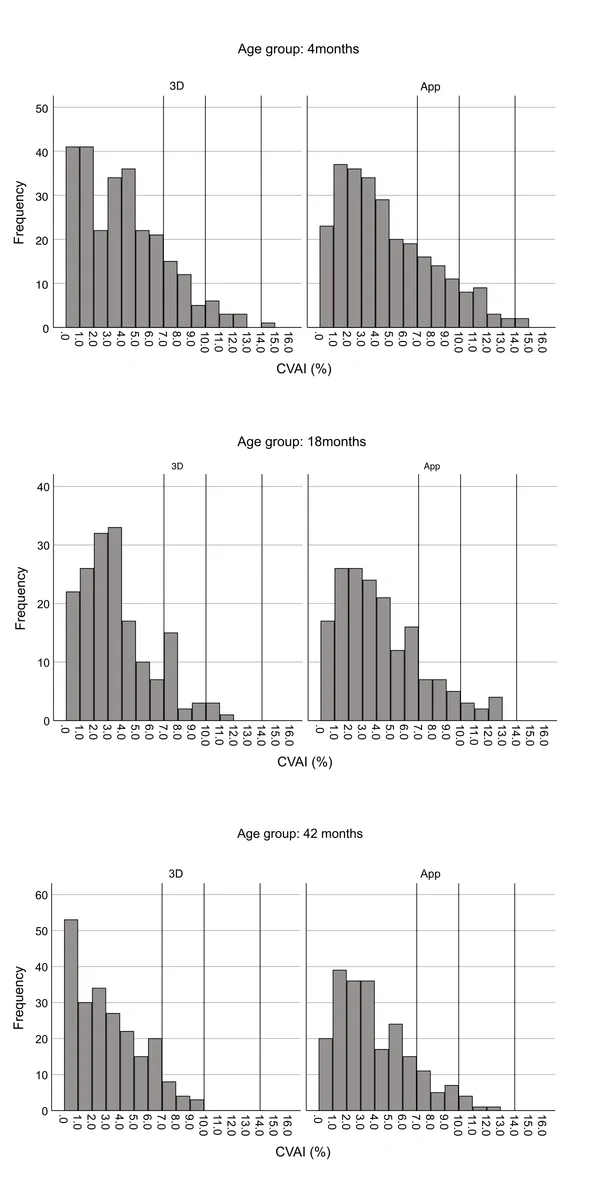
Distribution of CVAI (%) by age group and measurement method (3D scanner vs mobile app). CVAI was calculated using the shorter diagonal as the denominator ([CVAI(S), where S denotes the shorter diagonal]). Vertical reference lines indicate CVAI severity thresholds, marking the cut-offs for moderate (≥7%), severe (≥10%), and very severe (≥14%) categories. CVAI: Cranial Vault Asymmetry Index.

Figure 3 shows the corresponding distributions of Cephalic Index (%), which were approximately unimodal and centered in the mid-to-high range; the app similarly showed a modest rightward shift (ie, higher Cephalic Index values) relative to the 3D scanner. For the 3D scanner, Cephalic Index values were predominantly distributed within the normal-to-brachycephalic range, whereas values in the dolichocephalic range were relatively infrequent.

**Figure 3.**
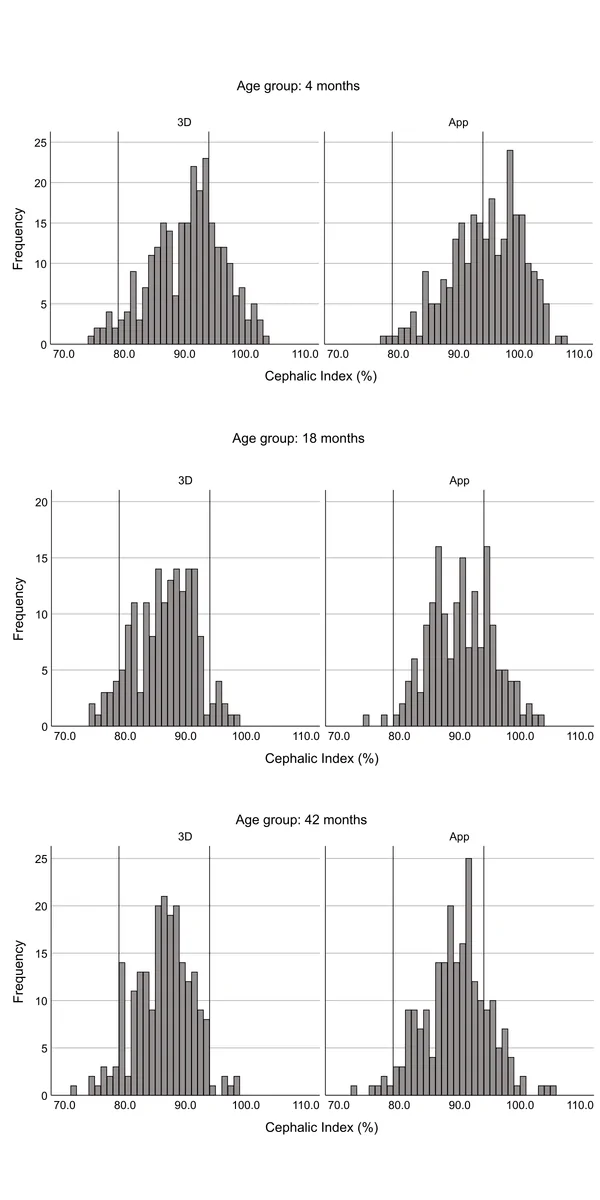
Distribution of Cephalic Index (%) by age group and measurement method (3D scanner vs mobile app). The 2 vertical reference lines indicate the prespecified Cephalic Index cut-off thresholds (79% and 94%), corresponding to the boundaries for dolichocephaly (<79%), normal (79%‐94%), and brachycephaly (>94%).

### Agreement Between the 3D Scanner and the Mobile App

Table 2 shows the agreement between the 3D scanner and the mobile app for CVAI and Cephalic Index at each assessment time point, and the corresponding Bland-Altman plots are presented in Figure 4 (CVAI) and Figure 5 (Cephalic Index).

For CVAI, at 4 months, the mean difference between the app and the scanner was small (0.14, SD 2.20), with 95% LoA from −4.18 to 4.46 and an ICC of 0.76 (95% CI 0.71‐0.81), indicating good agreement. At 18 months and 42 months, the mean differences for CVAI remained close to zero (0.32 and 0.31, respectively), but the LoAs were wider (−5.60 to 6.23 and −4.63 to 5.25, respectively), and the ICCs decreased to 0.42 (95% CI 0.29‐0.54) and 0.49 (95% CI 0.38‐0.59), suggesting poor-to-moderate agreement at these later time points.

For Cephalic Index, the app consistently yielded higher values than the scanner, with mean differences of 3.83, SD 3.42, 3.24, SD 3.07, and 2.61, SD 3.33 at 4, 18, and 42 months, respectively, indicating a systematic positive bias. The 95% LoA were wide at all time points (approximately −2.88 to 10.54 at 4 months, −2.77 to 9.25 at 18 months, and −3.91 to 9.14 at 42 months), indicating substantial individual-level variability. Although ICC point estimates were similar across age groups (0.69‐0.70), their 95% CI were wide, particularly at 4 and 18 months (4 months: 0.07‐0.88; 18 months: 0.09‐0.87; 42 months: 0.31‐0.84). Therefore, the ICC point estimates should not be overinterpreted as evidence of stable agreement for Cephalic Index. In this context, the Bland-Altman mean differences and LoA, which directly quantify the magnitude and direction of systematic bias and individual-level disagreement, provide a more clinically informative summary (Table 2 and Figure 5).

**Table 2.** Agreement between 3D scanner and mobile app-derived measurements of CVAI^a^ and Cephalic Index at each assessment time point.

* Measure	4 months (n=262)	18 months (n=171)^b^	42 months (n=216)
CVAI^c^			
Mean difference (app−scanner), mean (SD)	0.14 (2.20)	0.32 (3.02)	0.31 (2.52)
95% limits of agreement^d^	−4.18 to 4.46	−5.60 to 6.23	−4.63 to 5.25
ICC^e^ (95% CI)^f^	0.76 (0.71‐0.81)	0.42 (0.29‐0.54)	0.49 (0.38‐0.59)
Cephalic Index			
Mean difference (app−scanner), mean (SD)	3.83 (3.42)	3.24 (3.07)	2.61 (3.33)
95% LoA^d^^,^^g^	−2.88 to 10.54	−2.77 to 9.25	−3.91 to 9.14
ICC (95% CI)^f^	0.70 (0.07‐0.88)	0.69 (0.09‐0.87)	0.70 (0.31‐0.84)

a CVAI: Cranial Vault Asymmetry Index.

b At 18 months, 1 participant was excluded from CI-based analyses because of missing Cephalic Index data (n=170).

c CVAI was calculated using the shorter diagonal as the denominator [CVAI(S)].

d Limits of agreement were calculated as mean difference±1.96× SD of the differences.

e ICC: intraclass correlation coefficients.

f Intraclass correlation coefficients were calculated using a two-way mixed-effects model with absolute agreement, single measures.

g LoA: limits of agreement.

To assess heteroscedasticity in Bland-Altman differences, we examined Spearman correlations between the absolute method differences and the measurement magnitude (defined as the mean of the two methods). For CVAI, absolute differences increased modestly with increasing measurement magnitude across all ages (ρ=0.18‐0.29; *P*≤.004), suggesting mild heteroscedasticity. In contrast, for Cephalic Index, the association was weak and not consistently observed across age groups (ρ=0.06‐0.18), indicating no clear consistent pattern of heteroscedasticity across age groups.

**Figure 4.**
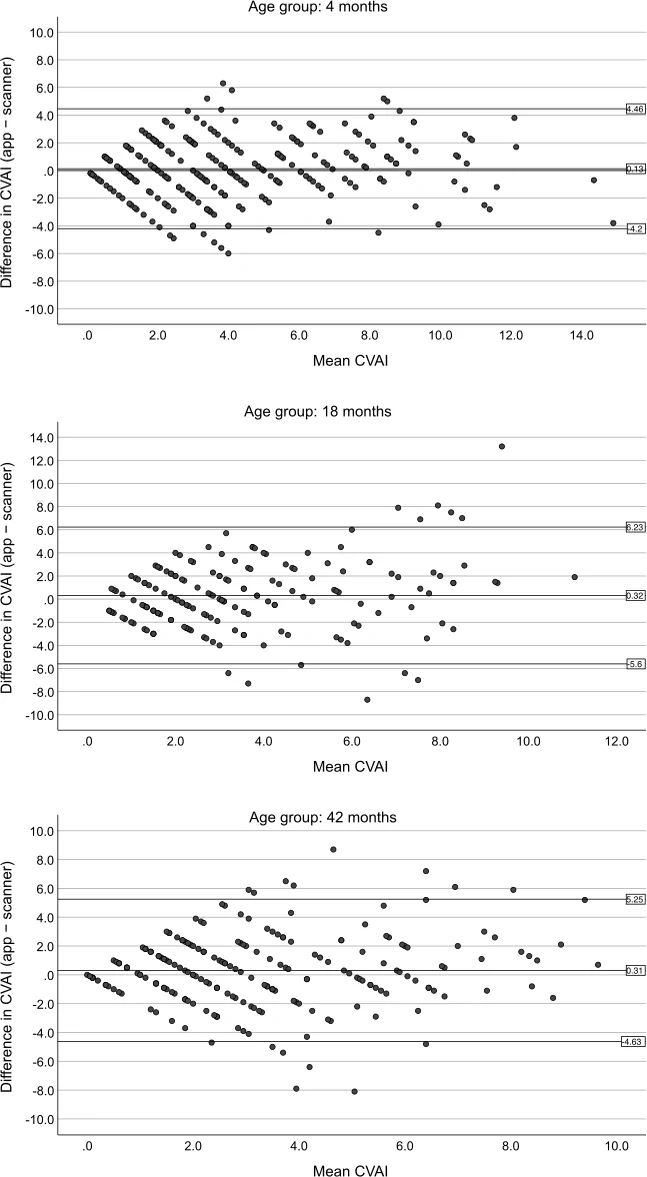
Bland-Altman plot of agreement between 3D scanner–derived and mobile app–derived CVAI measurements. CVAI: Cranial Vault Asymmetry Index.

**Figure 5.**
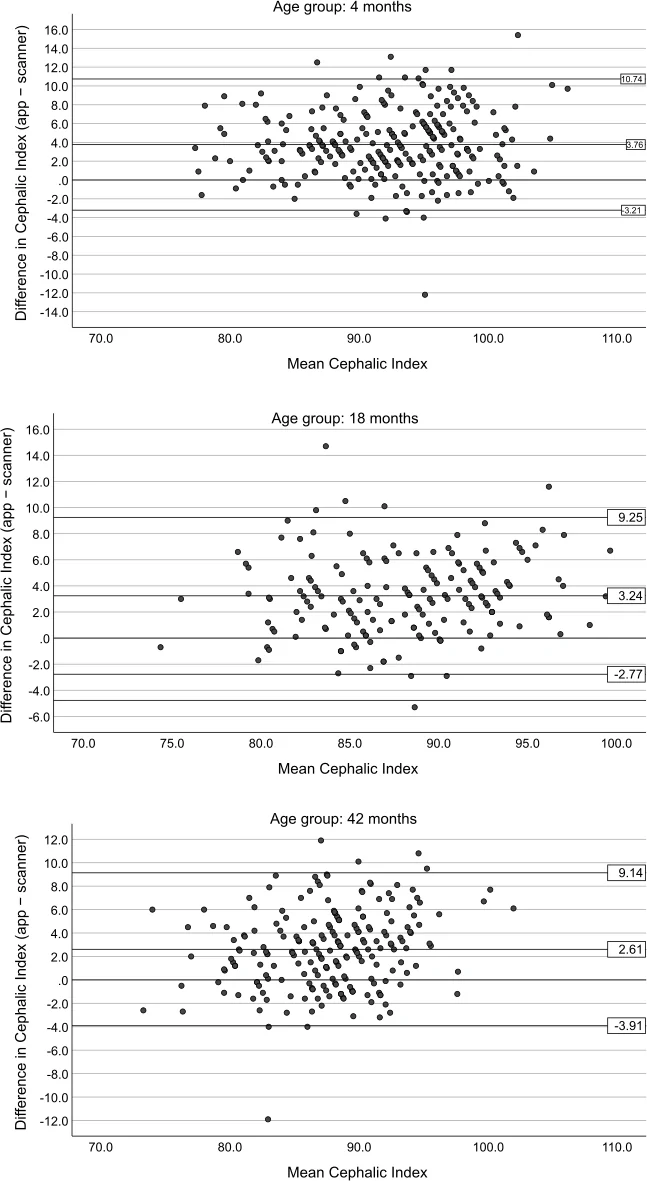
Bland-Altman plot of agreement between 3D scanner–derived and mobile app–derived Cephalic Index measurements.

### Threshold-Based Screening Performance

Table 3 summarizes scanner-defined prevalence and screening performance, including sensitivity, specificity, PPV, and NPV, when the same clinical thresholds were applied to both the 3D scanner (reference standard) and the mobile app. Specifically, for plagiocephaly (CVAI≥7%), sensitivity was 84.8% (39/46) at 4 months and decreased to 50.0% (12/24) and 53.3% (8/15) at 18 and 42 months, respectively, whereas specificity remained stable at 88.0% (190/216), 88.4% (130/147), and 89.6% (180/201). Scanner-defined prevalence decreased from 17.6% (46/262) at 4 months to 14.0% (24/171) and 6.9% (15/216) at 18 and 42 months, respectively, with PPV decreasing correspondingly from 60.0% (95% CI 47.9‐71.0) to 41.4% (95% CI 25.5‐59.3) and 27.6% (95% CI 14.7‐45.7); NPV remained high at 96.4% (190/197), 91.5% (130/142), and 96.3% (180/187).

**Table 3.** Screening performance of the mobile app relative to the 3D scanner reference standard.

Age	Prevalence, n/N (%)	Sensitivity, n/N (%)	95% CI	Specificity, (n/N) %	95% CI	PPV^a^, % (95% CI)	NPV^b^, % (95% CI)
Plagiocephaly^c^							
4 months	46/262 (17.6)	39/46 (84.8)	71.8‐92.4	(190/216) 88.0	82.9‐91.7	60.0 (47.9‐71.0)	96.4 (92.8‐98.3)
18 months	24/171 (14.0)	(12/24) 50.0	(31.4‐68.6)	(130/147) 88.4	82.3‐92.7	41.4 (25.5‐59.3)	91.5 (85.8‐95.1)
42 months	15/216 (6.9)	(8/15) 53.3	(30.1‐75.2)	(180/201) 89.6	84.6‐93.1	27.6 (14.7‐45.7)	96.3 (92.5‐98.2)
Brachycephaly^d^^,^^e^							
4 months	70/262 (26.7)	(64/70) 91.4	(82.5‐96.0)	(121/192) 63.0	56.0‐69.5	47.4 (39.2‐55.8)	95.3 (90.1‐97.8)
18 months	10/170 (5.9)	(10/10) 100.0	(72.2‐100.0)	(138/160) 86.3	80.1‐90.7	31.3 (18.0‐48.6)	100.0 (97.3‐100.0)
42 months	6/216 (2.8)	(6/6) 100.0	(61.0‐100.0)	(184/210) 87.6	82.5‐91.4	18.8 (8.9‐35.3)	100.0 (98.0‐100.0)
Dolichocephaly^d^^,^^f^							
4 months	11/262 (4.2)	(2/11) (5.1‐47.7)	(5.1‐47.7)	(251/251) 100.0	98.5‐100.0	100.0 (34.2‐100.0)	96.5 (93.6‐98.2)
18 months	13/170 (7.6)	(2/13) 15.4	(4.3‐42.2)	(157/157) 100.0	97.6‐100.0	100.0 (34.2‐100.0)	93.5 (88.7‐96.3)
42 months	12/216 (5.6)	(5/12) 41.7	(19.3‐68.0)	(203/204) 99.5	97.3‐99.9	83.3 (43.6‐97.0)	96.7 (93.3‐98.4)

a PPV: positive predictive value.

b NPV: negative predictive value.

c Cranial Vault Asymmetry Index (CVAI) was calculated using the shorter diagonal as the denominator [CVAI(S)]. Plagiocephaly was defined as CVAI≥7% for both the 3D scanner and the mobile app.

d At 18 months, 1 participant was excluded from brachycephaly and dolichocephaly analyses because of missing Cephalic Index data (n=170).

e Brachycephaly was defined as Cephalic Index>94% for both the 3D scanner and the mobile app.

f Dolichocephaly was defined as Cephalic Index<79% for both the 3D scanner and the mobile app.

For brachycephaly (Cephalic Index>94%), sensitivity was high across age groups (64/70, 91.4%, 10/10, 100.0% , and 6/6, 100.0% at 4, 18, and 42 months, respectively), while specificity increased from 63.0% (121/192) at 4 months to 86.3% (138/160) and 87.6% (184/210) at 18 and 42 months. Scanner-defined prevalence decreased from 26.7% (70/262) at 4 months to 5.9% (10/170) and 2.8% (6/216) at 18 and 42 months, respectively, accompanied by a decrease in PPV from 47.4% (64/135; 95% CI 39.2‐55.8) to 31.3% (10/32; 95% CI 18.0‐48.6) and 18.8% (6/32; 95% CI 8.9‐35.3), while NPV remained high at 95.3% (121/127)‐100.0% (184/184).

For dolichocephaly (Cephalic Index<79%), specificity remained≥99.5% across age groups, whereas sensitivity was low overall at 18.2% (2/11), 15.4% (2/13), and 41.7% (5/12) at 4, 18, and 42 months, respectively. PPV was 100.0% (2/2) at 4 and 18 months and 83.3% (5/6) at 42 months, whereas NPV ranged from 93.5% (157/168) to 96.7% (203/210).

As some Cephalic Index–based subgroup estimates were based on small numbers of scanner-positive cases, these sensitivity estimates—particularly those that appear high (eg, 100%)—should be interpreted cautiously. One 18-month participant with missing Cephalic Index data was excluded from the brachycephaly and dolichocephaly analyses only.

### Secondary Exploratory ROC Analyses of Continuous App-Based Indices

These ROC analyses were conducted as secondary exploratory analyses to describe the discriminative characteristics of the continuous app-based indices and should be interpreted as supplementary to the primary clinically anchored same-threshold screening analyses shown in Table 3.

For scanner-defined moderate-or-worse plagiocephaly, ROC analyses showed excellent discrimination at 4 months, with lower discriminative performance at 18 and 42 months. ROC-derived cut-off values varied across age groups, and sensitivity-prioritized cutoffs at older ages required substantial reductions in specificity, reinforcing the primary same-threshold findings that screening performance was most favorable at 4 months.

For brachycephaly, ROC analyses suggested apparently high discriminative performance at 18 and 42 months; however, these estimates were based on very small numbers of scanner-positive cases and should therefore be interpreted with caution as exploratory and potentially unstable. For dolichocephaly, ROC analyses also suggested good discrimination in some age groups, but the limited number of positive cases restricted the precision and interpretability of these estimates. Detailed ROC results, including AUCs, Youden-optimal cut-off values, and sensitivity-prioritized cut-off values, are provided in Multimedia Appendices 2-4.

### Agreement in Severity Classification

Additional analyses compared severity classifications derived from the 3D scanner and mobile app. Across age groups, discordance in 5-category CVAI-based plagiocephaly severity classification more often reflected overclassification by the app (App>3D scanner) than underclassification (App<3D scanner), and disagreements often occurred between adjacent severity categories. At 42 months, the observed severity range was more restricted, resulting in a 3×4 classification matrix (Multimedia Appendices 5-7). The overall direction of discordance is summarized in Multimedia Appendix 8, showing that discordance was more often in the direction of higher severity classification by the app.

Similarly, for 3-category Cephalic Index–based head-shape classifications (dolichocephaly, normal, or brachycephaly), discordance was predominantly in the direction of higher Cephalic Index–based classification, particularly toward brachycephaly, with minimal underclassification and no evidence of extreme misclassification across nonadjacent categories (Multimedia Appendices 9-11). The direction of discordance is summarized in Multimedia Appendix 12. At 18 months, 1 participant with missing Cephalic Index data was excluded from these analyses (valid n=170).

### Device-Stratified Sensitivity Analyses

Device-stratified analyses showed some variation across devices (Multimedia Appendix 13). For CVAI, the mean difference was small with both devices, but the direction differed at 18 months (iPad: 0.49, SD 3.13 vs Lenovo: −0.55, SD 2.23); the Lenovo subgroup was small (n=28). For Cephalic Index, positive bias was observed with both devices but was larger during the iPad period (eg, at 4 months: 4.24, SD 3.45 vs 3.06, SD 3.25). Device-stratified screening performance also varied by device and age (Multimedia Appendix 14), although the overall pattern of higher plagiocephaly sensitivity at 4 months was observed with both devices. Several subgroup estimates, particularly at later ages, were based on few scanner-positive cases and should be interpreted cautiously.

## Discussion

### Main Findings

The main finding of this study was that, under standardized, trained-examiner municipal health checkup conditions, app-based CVAI showed the most favorable screening performance for scanner-defined moderate-or-worse plagiocephaly at 4 months, whereas its performance was less favorable and more variable at 18 and 42 months. This age-related pattern was consistent across both agreement and screening performance analyses. Although ICCs provided a global summary index of agreement between methods, the Bland-Altman LoA were more clinically relevant for assessing individual-level disagreement and potential interchangeability between the app and the 3D scanner [24]. However, even at 4 months, the 95% LoA (−4.18 to 4.46) indicate that app-based values could still differ from scanner-based values by several percentage points in either direction. Importantly, small average differences between methods did not necessarily translate into stable classification at clinically relevant thresholds. When values were near the 7% threshold for moderate-or-worse plagiocephaly, even modest between-method differences could shift a child across the classification boundary. Accordingly, the app may be useful as a practical screening aid in structured, examiner-administered settings, but it should not be regarded as a direct substitute for 3D scanner–based classification when making decisions for individual children.

For Cephalic Index, a distinct pattern emerged. Across all ages, the app showed a consistent positive bias (ie, higher Cephalic Index values relative to the 3D scanner) and wide LoA, indicating a tendency toward systematic overestimation with substantial variability at the individual level. This may have contributed to increased false-positive classifications for brachycephaly, particularly at lower specificity levels. In addition, the Cephalic Index–based findings should not be interpreted as establishing robust age-specific screening thresholds, but rather viewed as exploratory signals that require confirmation in larger samples with sufficient numbers of scanner-positive cases. Device-stratified analyses also showed some variation across devices, including a reversal in the small CVAI bias at 18 months and differences in Cephalic Index bias. However, sequential device use was confounded with study period and accumulated examiner experience, precluding attribution to hardware alone.

### Comparison With Existing Literature

Prior validation work has largely been conducted under more controlled conditions than routine municipal health checkups. For example, Nakahari et al [8] evaluated the same app used in this study against a reference method using 3D-printed head models under highly standardized imaging conditions and proposed ROC-based cutoffs. In contrast, our study assessed Japanese infants and young children in real-world municipal health checkup settings, where unavoidable factors (eg, movement, hair, and variability in landmark visibility and camera-to-head distance) may introduce additional measurement variability. This difference in study context is important when interpreting the observed performance patterns, particularly the finding that agreement and screening performance for CVAI were most favorable at 4 months and declined at later ages in our data.

Given this context, our findings both align with and extend existing evidence in clinically relevant ways. In exploratory ROC analyses, the Youden-optimal cutoff for detecting scanner-defined moderate-or-worse plagiocephaly at 4 months was similar to that reported previously, suggesting convergent validity under comparable age conditions. However, these ROC-derived cutoffs should be interpreted as sample-specific exploratory findings rather than as proposed clinical thresholds for implementation [8]. For Cephalic Index–based outcomes, the ROC-derived cutoffs varied across ages and measurement contexts and should not be interpreted as establishing robust generalizable age-specific screening thresholds, particularly given the small number of scanner-positive cases in some subgroups. In addition, app-based Cephalic Index values consistently exceeded scanner-based values across all ages, indicating a systematic tendency toward overestimation. Correspondingly, ROC-derived app cutoffs to reproduce scanner-defined brachycephaly (Cephalic Index>94%) were substantially higher than those reported in prior studies, suggesting that Cephalic Index thresholds may not be directly transferable across measurement contexts without local calibration. Consistent with the primary same-threshold analyses, at 18 and 42 months, achieving high sensitivity required substantial reductions in specificity, indicating that cutoff adjustment alone may not fully compensate for age-related limitations in measurement agreement beyond early infancy. This study also adds an implementation-oriented perspective by evaluating “same-threshold” use (ie, applying identical clinical thresholds to both the app and the scanner) and by describing the direction and magnitude of discordance in severity classification. These analyses demonstrate how acceptable agreement for continuous indices may not translate directly into consistent threshold-based classification, thereby highlighting the importance of considering both measurement agreement and classification behavior in real-world screening pathways.

### Interpretation of Age-Related and Cephalic Index–Related Differences

#### Plagiocephaly

Agreement and discrimination of CVAI were strongest at 4 months but attenuated at 18 and 42 months, with corresponding reductions in sensitivity for detecting scanner-defined CVAI≥7%. This age-related pattern should be interpreted cautiously as a hypothesis-generating observation rather than a directly tested causal explanation. One possible contributor is a shift in case mix over time: CVAI may peak in early infancy and gradually improve as infants develop gross motor skills, which could shift the population distribution toward milder asymmetry at older ages [10,25,26]. In our study, the number of children with scanner-defined moderate-or-worse plagiocephaly was also smaller at older ages, particularly at 42 months, which increases statistical uncertainty in sensitivity estimates. This interpretation is consistent with the observed concentration of CVAI values in the lower range at older ages and with discordance often occurring between adjacent severity categories.

A second, nonmutually exclusive explanation relates to increased measurement difficulty under real-world image capture conditions. App-based CVAI estimation depends on accurate visualization and manual specification of key anatomical landmarks from a single vertex photograph; the app instructs users to capture an overhead image with the nose and ears visible and to annotate these landmarks, and prior validation work suggests that landmark visibility and positioning conditions can influence screening sensitivity [8]. Moreover, because prior validation studies were conducted using stationary head models without hair, performance in older infants and young children may be more susceptible to real-world capture challenges, including movement, hair coverage, and variable head positioning, which could increase landmark identification error and contour-tracing variability and reduce measurement repeatability in single-image assessments [8,27]. Our data could not determine whether the lower apparent performance at older ages was primarily attributable to changes in case mix (ie, fewer scanner-positive cases and a higher proportion of borderline values near classification thresholds) or to greater measurement difficulty under real-world conditions.

In addition to biological or case-mix differences across ages, part of the apparent age-related pattern may also reflect methodological features inherent to the measurement process. Because the 3D scanner was always used first, followed by app-based measurement by the same examiner with the same cap in place and minimal delay, the index test was performed under a partially shared measurement context with a fixed testing sequence. This design likely enhanced comparability between methods, but it may also have introduced the potential for overestimation of agreement and screening performance compared with fully independent assessments. Therefore, the observed age-related differences should not be interpreted solely as intrinsic differences in app performance across developmental stages, but rather in the context of potentially interacting biological, behavioral, and methodological factors.

#### Cephalic Index–Related Findings: Brachycephaly and Dolichocephaly

Across all assessment time points, the app tended to yield systematically higher Cephalic Index values than the 3D scanner, indicating a consistent positive bias. This shift may have contributed to the comparatively lower specificity for brachycephaly (scanner-defined Cephalic Index>94%) and to an increased frequency of false-positive classifications. This finding can be considered in relation to a previous smartphone-based validation study [28]. Kocabalkanli et al [28] compared smartphone-based cranial measurements with 3D reference measurements in 71 infants and reported minimal Bland-Altman bias for both CVAI (−0.05; 95% LoA, −3.85 to 3.76) and Cephalic Index (0.08; 95% LoA, −4.18 to 4.34). Although differences in the reference systems, study populations, app, and image-capture protocols preclude direct comparison, the CVAI agreement observed in their study was similar to that observed in our 4-month cohort (mean difference, 0.14; 95% LoA, −4.18 to 4.46), whereas our Cephalic Index showed a substantially larger positive bias (mean difference 3.83, 95% LoA −2.88 to 10.54). This index-specific contrast suggests that the positive Cephalic Index bias observed in our study may not be attributable to 2-dimensional image acquisition alone. Kocabalkanli et al [28] noted the importance of positioning the camera orthogonally to the measurement plane for smartphone-based cranial measurements. Potential contributors in our study include deviation of the camera from an orientation orthogonal to the cranial measurement plane, variation in head position during image acquisition, cranial contour extraction, the algorithm used to derive Cephalic Index from the 2-dimensional image, and differences between dimensions derived from a 2-dimensional cranial contour and those measured on a defined cross-section of a 3D model. In particular, differences in the estimation of cranial width or anteroposterior length could systematically affect Cephalic Index because Cephalic Index is calculated as the ratio of these dimensions. However, the app did not output the component cranial width and anteroposterior length measurements, and these potential mechanisms could therefore not be directly evaluated in the present study.

In contrast to brachycephaly, the app showed high specificity but low sensitivity for dolichocephaly (scanner-defined Cephalic Index<79%), indicating that scanner-defined cases of dolichocephaly were frequently missed by the app. This pattern may partly reflect the underlying distribution of Cephalic Index in the study population: because Cephalic Index varies with age in Japanese children, relatively few children fell below the 79% threshold, resulting in few positive cases and limited precision in sensitivity estimates [22]. In addition, the systematic positive bias in app-based Cephalic Index values may have shifted some measurements above the lower Cephalic Index threshold, thereby contributing to the low sensitivity for dolichocephaly.

Taken together, these features also help explain the uncertainty in agreement estimates for Cephalic Index. Although ICCs for Cephalic Index were approximately 0.69‐0.70, their 95% CIs were relatively wide, likely reflecting restricted between-child variability in Cephalic Index within this community-based sample. Moreover, the systematic positive bias indicates that disagreement between methods was not purely random. Therefore, for Cephalic Index, Bland-Altman mean differences and LoA provide a more transparent summary of the clinically relevant disagreement than ICCs alone.

#### Clinical Implications

Clinically, the potential role of the app appears to vary by age and clinical context. At 4 months—when early identification can guide timely counseling and referral—the app may be most useful as a screening aid when administered by a trained examiner within a structured health checkup setting. In contrast, at 18 and 42 months, agreement and/or screening performance were reduced, suggesting more limited utility for threshold-based clinical decision-making. At 42 months in particular, assessment should be interpreted as descriptive of residual cranial shape and supportive of parental reassurance and counseling, rather than as determinative of treatment eligibility. In practice, because this app is commonly used in home settings, parents may use its outputs to decide whether to seek specialist evaluation [8]. This timing is clinically important because helmet therapy outcomes are strongly age-dependent: a review recommends initiation before 6 months, reports that treatment is generally effective up to 12 months, and notes that the effect becomes limited after 12 months and poor after 18 months [2]. In addition, the Congress of Neurological Surgeons guideline recommends helmet therapy for infants with persistent moderate-to-severe plagiocephaly after a course of conservative treatment, and also for infants with moderate-to-severe deformity who present at an older age [29]. Consistent with these recommendations, delayed identification and initiation of treatment may reduce the achievable degree of correction, as older infants are less responsive to helmet therapy [7]. Therefore, incorporating app-based CVAI screening into the 4-month municipal health-checkup workflow could be pragmatically valuable, as this represents a near-universal point of contact and may facilitate early identification and timely referral for confirmatory assessment when moderate asymmetry is suspected.

At the same time, the LoA suggest that app-based measurements should not be considered directly interchangeable with 3D scanner–based classifications for individual clinical decision-making. Rather, the app appears more suitable for screening purposes—specifically, **i**dentifying children who may warrant confirmatory assessment**,** particularly when values lie, near clinically relevant thresholds, where classification may change according to the measurement method. For implementation, clear and practical operational guidance is essential to reduce avoidable variability, including maintaining a consistent camera-to-head distance, avoiding head tilt or rotation**,** and ensuring adequate visualization of key landmarks in a vertex-view photograph (ie, the nose and both ears), alongside adherence to the in-app measurement guide [8].

Finally, because app-based Cephalic Index values tended to be systematically higher than scanner-based values across age groups in this study, app-positive brachycephaly findings should be interpreted cautiously. For moderate-or-worse plagiocephaly, PPV decreased from 60.0% (39/65) at 4 months to 27.6% (8/29) at 42 months as scanner-defined prevalence declined; similarly, PPV for brachycephaly decreased from 47.4% (64/135) to 18.8% (6/32). Thus, particularly at later ages, app-positive findings may lead to additional confirmatory assessments for children who would not meet the corresponding 3D scanner–based threshold. Accordingly, positive findings at later ages require cautious interpretation in light of age, clinical context, and, when appropriate, confirmatory evaluation.

### Strengths and Limitations

This study has several strengths. First, measurements were obtained during municipal health checkups with broad population coverage, supporting the relevance of the findings to community-based screening in Japan. Second, app-based measurements were compared with a clinically used 3D scanner. Third, age-stratified analyses allowed assessment of agreement and screening performance across 3 developmental stages.

Several limitations should also be noted. First, findings may not generalize beyond the devices and OSs evaluated. Because the devices were used sequentially rather than concurrently, device type was confounded with study period and accumulated examiner experience, precluding separation of hardware effects from examiner learning or other temporal factors. Second, some subgroup analyses included very small numbers of scanner-positive cases, particularly in the 18-month Lenovo subgroup and at higher-severity plagiocephaly thresholds (CVAI≥10% and≥14%), resulting in potentially unstable estimates. Third, although JPMC technical staff were blinded to the app-based measurement results, JPMC—the developer of the index app—also performed postacquisition processing and quantitative analysis of the 3D scanner data. Therefore, the reference-standard analysis was not fully independent of the app developer and may have introduced potential bias. Fourth, although the examiner did not have access to the final quantitative results derived from the 3D scanner at the time of app-based measurement, both measurements were performed during the same visit by the same trained examiner in a fixed sequence (3D scanner first, app second) under a partially shared measurement context. This partial nonindependence may have favored agreement, and the observed performance may therefore represent an upper-bound estimate under standardized, trained-examiner conditions. Fifth, the app was not evaluated under its intended unsupervised home-use conditions; performance may differ when measurements are performed by untrained users. Sixth, repeated measurements were not collected, precluding assessment of intra- and interrater reproducibility and repeatability. Finally, ROC-derived cutoffs were sample-based point estimates without CIs, limiting the precision and generalizability of these exploratory findings.

### Conclusion

App-based CVAI showed the most favorable agreement and screening performance at 4 months under trained-examiner municipal health-checkup conditions, but performance was lower at 18 and 42 months. App-based Cephalic Index was systematically higher than scanner-based Cephalic Index, potentially increasing false-positive classifications for brachycephaly when identical thresholds are applied. Performance also varied to some extent between devices, although device effects could not be separated from study period and accumulated examiner experience. Overall, positive findings from the app—particularly beyond early infancy and for Cephalic Index-based measures—should be interpreted in light of the child’s age and broader clinical findings, with further evaluation or specialist referral considered only when clinically indicated, rather than treated as definitive diagnostic classifications.

## Supplementary material

10.2196/105473 Multimedia Appendix 1 Example screen of the mobile app used for cranial shape assessment. The app outputs “Yugami-do” (degree of asymmetry) and “Tantou-do” (degree of brachycephaly), which were operationally analyzed as app-based CVAI and app-based Cephalic Index, respectively.

10.2196/105473 Multimedia Appendix 2 Receiver operating characteristic (ROC) analysis of app-based CVAI to detect moderate-or-worse plagiocephaly defined by 3D scanner CVAI ≥ 7%, stratified by age group.

10.2196/105473 Multimedia Appendix 3 Receiver operating characteristic (ROC) analysis of app-based Cephalic Index to detect brachycephaly (3D scanner Cephalic Index > 94%), stratified by age group.

10.2196/105473 Multimedia Appendix 4 Receiver operating characteristic (ROC) analysis of app-based Cephalic Index to detect dolichocephaly (3D scanner Cephalic Index < 79%), stratified by age group.

10.2196/105473 Multimedia Appendix 5 Confusion matrix of 5-level plagiocephaly severity classification Cranial Vault Asymmetry Index (CVAI) between the 3D scanner and the mobile app at 4 months (n=262).

10.2196/105473 Multimedia Appendix 6 Confusion matrix of 5-level plagiocephaly severity classification Cranial Vault Asymmetry Index (CVAI) between the 3D scanner and the mobile app at 18 months (n=171).

10.2196/105473 Multimedia Appendix 7 Confusion matrix of 5-level plagiocephaly severity classification Cranial Vault Asymmetry Index (CVAI) between the 3D scanner and the mobile app at 42 months (n=216).

10.2196/105473 Multimedia Appendix 8 Direction of discordance between app- and 3D scanner–based 5-level plagiocephaly severity classifications (App < 3D scanner vs App > 3D scanner), by age group.

10.2196/105473 Multimedia Appendix 9 Confusion matrix of 3-category Cephalic Index-based head-shape classification between the 3D scanner and the mobile app at 4 months (n=262).

10.2196/105473 Multimedia Appendix 10 Confusion matrix of 3-category Cephalic Index-based head-shape classification between the 3D scanner and the mobile app at 18 months (valid n=170).

10.2196/105473 Multimedia Appendix 11 Confusion matrix of 3-category Cephalic Index-based head-shape classification between the 3D scanner and the mobile app at 42 months (n=216).

10.2196/105473 Multimedia Appendix 12 Direction of discordance between app- and 3D scanner–based 3-category Cephalic Index classifications (App < 3D scanner vs App > 3D scanner), by age group.

10.2196/105473 Multimedia Appendix 13 Device-stratified agreement between the 3D scanner and mobile app measurements of Cranial Vault Asymmetry Index (CVAI) and Cephalic Index at each assessment time point.

10.2196/105473 Multimedia Appendix 14 Device-stratified sensitivity and specificity of the mobile app using the 3D scanner as the reference standard.

10.2196/105473 Checklist 1 Completed STROBE checklist for cross-sectional studies.

10.2196/105473 Checklist 2 The STARD reporting checklist.

## Abbreviations

AUC: area under the curve
CA: cranial asymmetry
CT: computed tomography
CVAI: Cranial Vault Asymmetry Index
CVAI(S): Cranial Vault Asymmetry Index-Shorter Diagonal
ICC: intraclass correlation coefficient
JPMC: Japan Medical Company
LoA: limits of agreement
NPV: negative predictive value
PPV: positive predictive value
ROC: receiver operating characteristic
STARD: Standards for Reporting Diagnostic Accuracy Studies
STROBE: Strengthening the Reporting of Observational Studies in Epidemiology
UMIN: University Hospital Medical Information Network

## References

[1] Aarnivala H, Vuollo V, Harila V (2016). The course of positional cranial deformation from 3 to 12 months of age and associated risk factors: a follow-up with 3D imaging. Eur J Pediatr.

[2] Jung BK, Yun IS (2020). Diagnosis and treatment of positional plagiocephaly. Arch Craniofac Surg.

[3] Klimo P, Lingo PR, Baird LC (2016). Congress of neurological surgeons systematic review and evidence-based guideline on the management of patients with positional plagiocephaly. Neurosurgery.

[4] Takamatsu A, Hikosaka M, Kaneko T, Mikami M, Kaneko A (2021). Evaluation of the molding helmet therapy for Japanese infants with deformational plagiocephaly. JMA J.

[5] Tsugu W, Hikosaka M, Takamatsu A, Kaneko T (2025). Serial changes in cranial asymmetry during helmet therapy for deformational plagiocephaly. J Craniofac Surg.

[6] Miyabayashi H, Nagano N, Kato R (2022). Reference values for cranial morphology based on three-dimensional scan analysis in 1-month-old healthy infants in Japan. Neurol Med Chir (Tokyo).

[7] Kajita H, Tanaka I, Komuro H, Nishimaki S, Kusakawa I, Sakamoto K (2024). Efficacy of cranial orthosis for plagiocephaly based on 2D and 3D evaluation. Arch Plast Surg.

[8] Nakahari A, Hosono S, Gomi A, Kusakawa I, Tanaka I (2025). Validating the accuracy of the ‘Measurement of Baby’s Head Shape mobile application. IEEJ Trans Electr Electron Eng.

[9] Seifabadi R, Aalamifar F, Hezaveh SH, Kocabalkanli C, Wilburn K, Linguraru MG (2021). Toward quantitative assessment of deformational plagiocephaly and brachycephaly at the point-of-care. J Med Imaging (Bellingham).

[10] Hutchison BL, Hutchison LAD, Thompson JMD, Mitchell EA (2004). Plagiocephaly and brachycephaly in the first two years of life: a prospective cohort study. Pediatrics.

[11] (2025). Overview of the FY2023 Regional Health and Health Promotion Project Report. Ministry of Health Labour and Welfare.

[12] Bossuyt PM, Reitsma JB, Bruns DE (2015). STARD 2015: an updated list of essential items for reporting diagnostic accuracy studies. BMJ.

[13] Elm E von, Altman DG, Egger M, Pocock SJ, Gøtzsche PC, Vandenbroucke JP (2007). Strengthening the reporting of observational studies in epidemiology (STROBE) statement: guidelines for reporting observational studies. BMJ.

[14] (2021). Maternal and child health handbook [Webpage in Japanese]. Children and Families Agency.

[15] About infant and toddler health checkups [Webpage in Japanese]. Children and Families Agency, Government of Japan.

[16] Imoto N, Hayashi M (2025). From handbooks to platforms: Japan’s maternal and child health services in the digital era. J Glob Health.

[17] Kato R, Nagano N, Sasano M, Sumi K, Morioka I (2025). Natural progression of cranial shape following helmet therapy for deformational plagiocephaly. J Clin Med.

[18] Tanaka Y, Miyabayashi H, Noto T, Kato R, Nagano N, Morioka I (2025). A novel prophylactic device against head deformity to prevent severe positional plagiocephaly. J Clin Med.

[19] Aihara Y, Komatsu K, Dairoku H, Kubo O, Hori T, Okada Y (2014). Cranial molding helmet therapy and establishment of practical criteria for management in Asian infant positional head deformity. Childs Nerv Syst.

[20] Noto T, Nagano N, Kato R (2021). Natural-course evaluation of infants with positional severe plagiocephaly using a three-dimensional scanner in Japan: comparison with those who received cranial helmet therapy. J Clin Med.

[21] Remobaby michigan [Webpage in Japanese]. Gunze Medical Japan Ltd.

[22] Koizumi T, Komuro Y, Hashizume K, Yanai A (2010). Cephalic index of japanese children with normal brain development. J Craniofac Surg.

[23] Koo TK, Li MY (2016). A guideline of selecting and reporting intraclass correlation coefficients for reliability research. J Chiropr Med.

[24] Bland JM, Altman DG (1999). Measuring agreement in method comparison studies. Stat Methods Med Res.

[25] Akutsu N, Koyama J, Kawamura A, Sasayama T (2024). Prevalence and severity of positional posterior plagiocephaly and positional posterior brachycephaly in children and adolescents in Japan. Neurol Med Chir (Tokyo).

[26] Narumi S, Honda Y, Kotoku Y (2026). Age-dependent changes in infant head shape: a smartphone app-based study in Japan. Clin Pediatr Endocrinol.

[27] Lim YC, Abdul Shakor AS, Shaharudin R (2021). Reliability and accuracy of 2D photogrammetry: a comparison with direct measurement. Front Public Health.

[28] Kocabalkanli C, Fenton R, Gaetani S, Aalamifar F, Linguraru MG, Seifabadi R (2025). Accuracy of cranial shape measurements using smartphones: a prospective study. Cleft Palate Craniofac J.

[29] Tamber MS, Nikas D, Beier A (2016). Congress of neurological surgeons systematic review and evidence-based guideline on the role of cranial molding orthosis (Helmet) therapy for patients with positional plagiocephaly. Neurosurgery.

