# Supplementary material for: Agreement and Screening Performance of an App-Based Cranial Shape Assessment Tool Compared With a 3D Scanner in Municipal Child Health Checkups in Japan: Instrument Validation Study
Source: JMIR Pediatr Parent. 2026 Oct 13;9:e105473. doi: 10.2196/105473 (PMC13662245; doi:10.2196/105473)
Supplement: Supplementary file 2 [file pediatrics-v9-e105473-s016.pdf]

Multimedia Appendix 2. The STARD reporting checklist.

For checking that diagnostic accuracy study articles can be understood and used by everyone

How to use this reporting checklist

This reporting checklist allows authors to demonstrate that their manuscripts adhere to the STARD reporting guideline.

If you have not used a reporting guideline before, read about how and why to use them and check whether STARD is the most applicable reporting guideline for your work.

Reporting guidelines are most useful when used early in research. When writing a manuscript or application, consider using the full guidance where you'll find explanations and examples for each item.

After writing, demonstrate adherence by completing this checklist:

1. Specify where each item is described (see Note 1).
2. Cite this checklist (See Note 2).
3. Include your completed checklist as a supplement when submitting to a journal so that future readers can use it to find information.

	Item Description	Location (or reason for not reporting)
**Title or abstract**		
1. Identification as a study of diagnostic accuracy	Identification as a study of diagnostic accuracy using at least one measure of accuracy (such as sensitivity, specificity, predictive values, or AUC).	Title (p1); Abstract, Methods and Results (pp1–2). The study is identified as evaluating screening performance, including sensitivity and specificity, against a 3D scanner reference standard; ROC/AUC analyses are also described in the Methods.
**Abstract**		
2. Abstract	Structured summary of study design, methods, results and conclusions (for specific guidance, see STARD for Abstracts).	Abstract (pp1–2). Structured summary of the study background, objective, methods, results, and conclusions.
**Introduction**		
3. Background	Scientific and clinical background, including the intended use and clinical role of the index test.	Introduction, Background (pp3–4). Scientific and clinical background, including the intended screening role of the mobile application, is described.
4. Objectives	Study objectives and hypotheses.	Introduction, final paragraph (p4). Study objectives are stated.
**Methods**		
5. Study design	Whether data collection was planned before the index test and reference standard were performed (prospective study) or after (retrospective study).	Methods, Study design and setting (pp4–5). The study was conducted as a cross-sectional study with prospectively collected data.
Participants		
6. Eligibility criteria	Eligibility criteria.	Methods, Participants (p5). Eligibility and exclusion criteria are described.
7. Identifying eligible participants	On what basis potentially eligible participants were identified (such as symptoms, results from previous tests, inclusion in registry).	Methods, Participants (p5). Potentially eligible participants were identified from children attending age-specific municipal health checkups in Goto City.
8. Setting, location, and dates	Where and when potentially eligible participants were identified (setting, location and dates).	Methods, Study design and setting; Methods, Participants (pp4–5). Study setting, locations, and study period are described.
9. Consecutive, random or convenience series	Whether participants formed a consecutive, random or convenience series.	Methods, Participants (p5). Participants were recruited from attendees at routine municipal health checkups with near-

		universal population coverage.
Test Methods		
10. Index test & Reference standard	10a. Index test<br><br>10b. Reference standard	Methods, Reference standard: 3D scanner (pp6–7); Methods, Index test: mobile application (pp7–8). The 3D scanner and mobile application are described.
11. Reference standard rationale	Rationale for choosing the reference standard (if alternatives exist).	Methods, Reference standard: 3D scanner (p6). The rationale for using the 3D scanner as the reference standard is described.
12. Index test and reference standard cut-offs or categories	12a. Definition of and rationale for test positivity cut-offs or result categories of the index test, distinguishing prespecified from exploratory.<br><br>12b. Definition of and rationale for test positivity cut-offs or result categories of the reference standard, distinguishing prespecified from exploratory.	Methods, Definitions of cranial indices and clinical thresholds (p9); Primary analysis: Screening performance and Secondary exploratory ROC analyses (pp10–11). Prespecified clinical thresholds and exploratory ROC-derived cutoffs are described.
13. Information available to performers or readers of the index test and reference standard assessors	13a. Whether clinical information and reference standard results were available to the performers or readers of the index test.<br><br>13b. Whether clinical information and index test results were available to the assessors of the reference standard.	Methods, Blinding and test interpretation (pp8–9). Information available to the index-test examiner and reference-standard assessors, including blinding and partial nonindependence, is described.
Analysis		
14. Analysis methods	Methods for estimating or comparing measures of diagnostic accuracy.	Methods, Statistical analysis (pp9–12). Methods for agreement, screening performance, ROC analyses, and sensitivity analyses are described.
15. Indeterminate results	How indeterminate index test or reference standard results were handled.	Methods, Data collection procedures (p6); Results, Participant characteristics (p13). Measurements were omitted when the child was excessively distressed or uncooperative, and failed 3D reconstructions were excluded from analysis.
16. Missing data	How missing data on the index test and reference standard were handled.	Methods, Statistical analysis (p10); Results, Participant characteristics (p13); Table 2 and Table 3 footnotes (pp17–20). Missing data were handled using paired measurements and listwise deletion; one 18-month participant with missing app-based CI data was excluded from CI-based analyses only.
17. Variability	Any analyses of variability in diagnostic accuracy, distinguishing prespecified from exploratory.	Methods, Secondary exploratory ROC analyses (p11); Methods, Sensitivity analyses: device-stratified analyses (p12). Variability was examined across age groups and device periods.
18. Intended sample size	Intended sample size and how it was determined.	Methods, Study design and setting (p5). No formal a priori sample size calculation was conducted; all eligible attendees during the study period were targeted for enrollment.
**Results**		
Participants		
19. Participant flow diagram	Flow of participants, using a diagram.	Results, Participant characteristics; Figure 1 (p13). Participant flow by age group is reported.
20. Baseline characteristics	Baseline demographic and clinical characteristics of participants.	Results, Participant characteristics (pp13–14); Table 1 (p14). Baseline demographic and clinical characteristics are reported by age group.
21a. Participants with and without the target	21a. Distribution of severity of disease in	Results, Distribution of cranial indices by age group and measurement method (pp14–16); Table 3 (pp19–20); severity/classification analyses (pp21–22). Severity distributions

condition	those with the target condition.<br><br>21b. Distribution of alternative diagnoses in those without the target condition	and classification results are reported; alternative diagnoses were not applicable.
22. Time interval	Time interval and any clinical interventions between index test and reference standard.	Methods, Data collection procedures (p6); Methods, Index test: mobile application and Blinding and test interpretation (pp8–9). Both tests were performed during the same visit, with minimal delay and no clinical intervention between tests.
Test Results		
23. Index test and reference standard results	Cross tabulation of the index test results (or their distribution) by the results of the reference standard.	Results, Threshold-based screening performance; Table 3 (pp19–21); Results, Secondary exploratory ROC analyses (p21); Multimedia Appendices 4–14. Index-test results relative to the reference standard are reported.
24. Estimates of accuracy	Estimates of diagnostic accuracy and their precision (such as 95% CIs).	Results, Threshold-based screening performance; Table 3 (pp19–21); Results, Secondary exploratory ROC analyses (p21); Multimedia Appendices 4–6. Estimates of screening accuracy and their precision are reported.
25. Adverse events	Any adverse events from performing the index test or the reference standard.	Methods, Data collection procedures (p6). Measurements were omitted if the child was excessively distressed or uncooperative; no test-related adverse events were reported.
**Discussion**		
26. Limitations	Study limitations, including sources of potential bias, statistical uncertainty and generalisability.	Discussion, Strengths and limitations (pp30–31). Study limitations, including potential sources of bias, statistical uncertainty, and generalizability, are discussed.
27. Implications for Practice	Implications for practice, including the intended use and clinical role of the index test.	Discussion, Clinical implications (pp28–30); Conclusion (p31). The intended clinical role of the application as a screening aid, with confirmatory assessment when indicated, is discussed.
**Other information**		
28. Registration	Registration number and name of registry.	Methods, Ethical considerations (p12). The study was prospectively registered in the UMIN Clinical Trials Registry (UMIN000052342).
29. Protocol	Where the full study protocol can be accessed.	Methods, Ethical considerations (p12). Key protocol information is available in the UMIN Clinical Trials Registry record (UMIN000052342).
30. Funding	Sources of funding and other support; role of funders.	Funding (p32). The source of funding and the funder's role are reported.

1 How to specify where content is

Tell the reader where they can find information. E.g.,

- Results; paragraph 2
- Methods, Participants; paragraphs 1 & 2.
- Table 3
- Supplement B, para. 4

If you have chosen not to describe an item, explain why. You can do this in the checklist, or as a note below it.

You can describe items in the article body, or in tables, figures, or supplementary materials, and should prioritize items you feel are most important to your intended audience. The order of items in your manuscript does not need to match the order of items in this checklist. You can decide how best to structure your work.

2 How to cite

Describe how you used STARD at the end of your Methods section, referencing the resources you used e.g.,

‘We used the STARD reporting guideline(1) to draft this manuscript, and the STARD reporting checklist(2) when editing, included in supplement A’

If you use a reporting checklist, remember to include it as a supplement when publishing so that readers can easily find information and see how you have interpreted the guidance.

1. Bossuyt PM, Reitsma JB, Bruns DE, Gatsonis CA, Glasziou PP, Irwig L, et al. STARD 2015: An updated list of essential items for reporting diagnostic accuracy studies. BMJ [Internet]. 2015 Oct;351:h5527. Available from: https://www.bmj.com/content/351/bmj.h5527

2. Bossuyt PM, Reitsma JB, Bruns DE, Gatsonis CA, Glasziou PP, Irwig L, et al. The STARD reporting checklist. In: Harwood J, Albury C, Beyer J de, Schlüssel M, Collins G, editors. The EQUATOR network reporting guideline platform [Internet]. The UK EQUATOR Centre; 2025. Available from: https:/resources.equator-network.org/reporting-guidelines/stard/stard-checklist.docx
