# Supplementary material for: Agreement and Screening Performance of an App-Based Cranial Shape Assessment Tool Compared With a 3D Scanner in Municipal Child Health Checkups in Japan: Instrument Validation Study
Source: JMIR Pediatr Parent. 2026 Oct 13;9:e105473. doi: 10.2196/105473 (PMC13662245; doi:10.2196/105473)
Supplement: Supplementary file 3 [file pediatrics-v9-e105473-s002.docx]

Multimedia Appendix 2. Receiver operating characteristic (ROC) analysis of app-based CVAI to detect moderate-or-worse plagiocephaly defined by 3D scanner CVAI ≥ 7%, stratified by age group.

| Age group | n (positive/negative) | AUC  (95% CI) | Youden-optimal cutoff  (app CVAI, %) | Sens  (%) | Spec  (%) | Youden’s index | Sensitivity-prioritized cutoff  (app CVAI, %)^a^ | Sens  (%) | Spec  (%) |
| --- | --- | --- | --- | --- | --- | --- | --- | --- | --- |
| 4 months | 262  (46/216) | 0.93  (0.89–0.97) | 5.5 | 93.5 | 81.0 | 0.75 | 5.5 | 93.5 | 81.0 |
| 18 months | 171  (24/147) | 0.77  (0.67–0.87) | 3.5 | 87.5 | 61.2 | 0.49 | 1.5 | 95.8 | 28.6 |
| 42 months | 216  (15/201) | 0.75  (0.59–0.92) | 5.5 | 66.7 | 83.1 | 0.50 | 0.5 | 93.3 | 9.5 |

^a^ The sensitivity-prioritized cutoff was defined as the highest cutoff that achieved sensitivity ≥0.90, thereby maximizing specificity under the sensitivity constraint.
