# Supplementary material for: Agreement and Screening Performance of an App-Based Cranial Shape Assessment Tool Compared With a 3D Scanner in Municipal Child Health Checkups in Japan: Instrument Validation Study
Source: JMIR Pediatr Parent. 2026 Oct 13;9:e105473. doi: 10.2196/105473 (PMC13662245; doi:10.2196/105473)
Supplement: Supplementary file 4 [file pediatrics-v9-e105473-s003.docx]

Multimedia Appendix 3. Receiver operating characteristic (ROC) analysis of app-based Cephalic Index to detect brachycephaly (3D scanner Cephalic Index > 94%), stratified by age group.

| Age group | n  (positive/negative) | AUC  (95% CI) | Youden-optimal cutoff  (app Cephalic Index, %) | Sens  (%) | Spec  (%) | Youden’s index | Sensitivity-prioritized cutoff  (app Cephalic Index, %)^a^ | Sens  (%) | Spec  (%) |
| --- | --- | --- | --- | --- | --- | --- | --- | --- | --- |
| 4 months | 262  (70/192) | 0.86  (0.81–0.90) | 95.0 | 84.7 | 70.3 | 0.56 | 94.5 | 90.4 | 63.0 |
| 18 months | 170  (10/160)^b^ | 0.96  (0.93–0.99) | 94.5 | 100 | 86.3 | 0.86 | 96.5 | 90 | 94.4 |
| 42 months | 216  (6/210) | 0.98  (0.96–1.00) | 96.5 | 100 | 94.8 | 0.95 | 96.5 | 100 | 94.8 |

^a^ The sensitivity-prioritized cutoff was defined as the highest cutoff that achieved sensitivity ≥0.90, thereby maximizing specificity under the sensitivity constraint.

^b^ One case had missing Cephalic Index data in the 18-month group and was excluded from the ROC analysis (valid n=170).

Estimates for the 18- and 42-month groups should be interpreted cautiously because they were based on very small numbers of scanner-positive cases.
