# Supplementary material for: Agreement and Screening Performance of an App-Based Cranial Shape Assessment Tool Compared With a 3D Scanner in Municipal Child Health Checkups in Japan: Instrument Validation Study
Source: JMIR Pediatr Parent. 2026 Oct 13;9:e105473. doi: 10.2196/105473 (PMC13662245; doi:10.2196/105473)
Supplement: Supplementary file 5 [file pediatrics-v9-e105473-s004.docx]

Multimedia Appendix 4. Receiver operating characteristic (ROC) analysis of app-based Cephalic Index to detect dolichocephaly (3D scanner Cephalic Index < 79%), stratified by age group.

| Age group | n  (positive/negative) | AUC  (95% CI) | Youden-optimal cutoff  (app Cephalic Index, %) | Sens  (%) | Spec  (%) | Youden’s index | Sensitivity-prioritized cutoff  (app Cephalic Index, %)^a^ | Sens  (%) | Spec  (%) |
| --- | --- | --- | --- | --- | --- | --- | --- | --- | --- |
| 4 months | 262  (11/251) | 0.97  (0.95–1.00) | 87.5 | 100 | 88.8 | 0.89 | 86.5 | 90.9 | 91.6 |
| 18 months | 170  (13/157)^b^ | 0.84  (0.75–0.94) | 88.5 | 92.3 | 63.1 | 0.55 | 88.5 | 92.3 | 63.1 |
| 42 months | 216  (12/204) | 0.95  (0.91–0.99) | 85.5 | 100 | 81.4 | 0.81 | 85.5 | 100 | 81.4 |

^a^ The sensitivity-prioritized cutoff was defined as the lowest cutoff that achieved sensitivity ≥0.90, thereby maximizing specificity under the sensitivity constraint, because positivity was defined as app Cephalic Index ≤ cutoff.

^b^ One case had missing Cephalic Index data in the 18-month group and was excluded from the ROC analysis (valid n=170).

Note: In this analysis, smaller app Cephalic Index values indicate stronger evidence of dolichocephaly, and positivity was defined as app Cephalic Index ≤ cutoff.
