# Supplementary material for: Agreement and Screening Performance of an App-Based Cranial Shape Assessment Tool Compared With a 3D Scanner in Municipal Child Health Checkups in Japan: Instrument Validation Study
Source: JMIR Pediatr Parent. 2026 Oct 13;9:e105473. doi: 10.2196/105473 (PMC13662245; doi:10.2196/105473)
Supplement: Supplementary file 6 [file pediatrics-v9-e105473-s005.docx]

Multimedia Appendix 5. Confusion matrix of 5-level plagiocephaly severity classification Cranial Vault Asymmetry Index (CVAI) between the 3D scanner and the mobile app at 4 months (n=262).

| 3D scanner＼App | Normal | Mild | Moderate | Severe | Very severe | Row total |
| --- | --- | --- | --- | --- | --- | --- |
| Normal | 142 (81.6) | 21 (12.1) | 11 (6.3) | 0 (0.0) | 0 (0.0) | 174 |
| Mild | 14 (33.3) | 13 (31.0) | 11 (25.6) | 4 (9.5) | 0 (0.0) | 42 |
| Moderate | 2 (6.3) | 4 (12.5) | 17 (53.1) | 9 (28.1) | 0 (0.0) | 32 |
| Severe | 0 (0.0) | 1 (8.3) | 2 (16.7) | 8 (66.7) | 1 (8.3) | 12 |
| Very severe | 0 (0.0) | 0 (0.0) | 0 (0.0) | 1 (50.0) | 1 (50.0) | 2 |
| Column total | 158 | 39 | 41 | 22 | 2 | 262 |

Values are n (row %).
