# Supplementary material for: Agreement and Screening Performance of an App-Based Cranial Shape Assessment Tool Compared With a 3D Scanner in Municipal Child Health Checkups in Japan: Instrument Validation Study
Source: JMIR Pediatr Parent. 2026 Oct 13;9:e105473. doi: 10.2196/105473 (PMC13662245; doi:10.2196/105473)
Supplement: Supplementary file 7 [file pediatrics-v9-e105473-s006.docx]

Multimedia Appendix 6. Confusion matrix of 5-level plagiocephaly severity classification Cranial Vault Asymmetry Index (CVAI) between the 3D scanner and the mobile app at 18 months (N=171).

| 3D scanner＼App | Normal | Mild | Moderate | Severe | Very severe | Row total |
| --- | --- | --- | --- | --- | --- | --- |
| Normal | 99 (76.2) | 19 (14.6) | 7 (5.4) | 4 (3.1) | 1 (0.8) | 130 |
| Mild | 7 (41.2) | 5 (29.4) | 4 (23.5) | 1 (5.9) | 0 (0.0) | 17 |
| Moderate | 5 (25.0) | 4 (20.0) | 8 (40.0) | 3 (15.0) | 0 (0.0) | 20 |
| Severe | 3 (75.0) | 0 (0.0) | 0 (0.0) | 1 (25.0) | 0 (0.0) | 4 |
| Column total | 114 | 28 | 19 | 9 | 1 | 171 |

Values are n (row %).
