# Supplementary material for: Agreement and Screening Performance of an App-Based Cranial Shape Assessment Tool Compared With a 3D Scanner in Municipal Child Health Checkups in Japan: Instrument Validation Study
Source: JMIR Pediatr Parent. 2026 Oct 13;9:e105473. doi: 10.2196/105473 (PMC13662245; doi:10.2196/105473)
Supplement: Supplementary file 8 [file pediatrics-v9-e105473-s007.docx]

Multimedia Appendix 7. Confusion matrix of 5-level plagiocephaly severity classification Cranial Vault Asymmetry Index (CVAI) between the 3D scanner and the mobile app at 42 months (N=216).

| 3D scanner＼App | Normal | Mild | Moderate | Severe | Row total |
| --- | --- | --- | --- | --- | --- |
| Normal | 134 (80.7) | 21 (12.7) | 9 (5.4) | 2 (1.2) | 166 |
| Mild | 10 (28.6) | 15 (42.9) | 8 (22.9) | 2 (5.7) | 35 |
| Moderate | 4 (26.7) | 3 (20.0) | 6 (40.0) | 2 (13.3) | 15 |
| Column total | 148 | 39 | 23 | 6 | 216 |

Note: Values are n (row %). At 42 months, “very severe” (CVAI ≥14%) did not occur in either method, and the 3D scanner had no “severe” cases; thus the matrix is 3×4 (scanner: normal–moderate; app: normal–severe). The highest observed category was moderate (3D scanner) and severe (app).
