# Supplementary material for: Agreement and Screening Performance of an App-Based Cranial Shape Assessment Tool Compared With a 3D Scanner in Municipal Child Health Checkups in Japan: Instrument Validation Study
Source: JMIR Pediatr Parent. 2026 Oct 13;9:e105473. doi: 10.2196/105473 (PMC13662245; doi:10.2196/105473)
Supplement: Supplementary file 9 [file pediatrics-v9-e105473-s008.docx]

Multimedia Appendix 8. Direction of discordance between app- and 3D scanner–based 5-level plagiocephaly severity classifications (App < 3D scanner vs App > 3D scanner), by age group.

| Age group | Under-classification  (App < scanner) | Exact agreement | Over-classification  (App > scanner) | Total |
| --- | --- | --- | --- | --- |
| 4 months | 24 (9.2) | 181 (69.1) | 57 (21.8) | 262 |
| 18 months | 19 (11.1) | 113 (66.1) | 39 (22.8) | 171 |
| 42 months | 17 (7.9) | 155 (71.8) | 44 (20.4) | 216 |

Values are n (row %).
