# Supplementary material for: Agreement and Screening Performance of an App-Based Cranial Shape Assessment Tool Compared With a 3D Scanner in Municipal Child Health Checkups in Japan: Instrument Validation Study
Source: JMIR Pediatr Parent. 2026 Oct 13;9:e105473. doi: 10.2196/105473 (PMC13662245; doi:10.2196/105473)
Supplement: Supplementary file 10 [file pediatrics-v9-e105473-s009.docx]

Multimedia Appendix 9. Confusion matrix of 3-category Cephalic Index-based head-shape classification between the 3D scanner and the mobile app at 4 months (n=262).

| 3D scanner＼App | Dolichocephaly | Normal | Brachycephaly | Row total |
| --- | --- | --- | --- | --- |
| Dolichocephaly | 2 (18.2) | 9 (81.8) | 0 (0.0) | 11 |
| Normal | 0 (0.0) | 110 (60.8) | 71 (39.2) | 181 |
| Brachycephaly | 0 (0.0) | 6 (8.6) | 64 (91.4) | 70 |
| Column total | 2 | 125 | 135 | 262 |

Values are n (row %).
