# Supplementary material for: Agreement and Screening Performance of an App-Based Cranial Shape Assessment Tool Compared With a 3D Scanner in Municipal Child Health Checkups in Japan: Instrument Validation Study
Source: JMIR Pediatr Parent. 2026 Oct 13;9:e105473. doi: 10.2196/105473 (PMC13662245; doi:10.2196/105473)
Supplement: Supplementary file 11 [file pediatrics-v9-e105473-s010.docx]

Multimedia Appendix 10. Confusion matrix of 3-category Cephalic Index-based head-shape classification between the 3D scanner and the mobile app at 18 months (n=170).

| 3D scanner＼App | Dolichocephaly | Normal | Brachycephaly | Row total |
| --- | --- | --- | --- | --- |
| Dolichocephaly | 2 (15.4) | 11 (84.6) | 0 (0.0) | 13 |
| Normal | 0 (0.0) | 125 (85.0) | 22 (15.0) | 147 |
| Brachycephaly | 0 (0.0) | 0 (0.0) | 10 (100.0) | 10 |
| Column total | 2 | 136 | 32 | 170 |

Values are n (row %).
