# Supplementary material for: Agreement and Screening Performance of an App-Based Cranial Shape Assessment Tool Compared With a 3D Scanner in Municipal Child Health Checkups in Japan: Instrument Validation Study
Source: JMIR Pediatr Parent. 2026 Oct 13;9:e105473. doi: 10.2196/105473 (PMC13662245; doi:10.2196/105473)
Supplement: Supplementary file 12 [file pediatrics-v9-e105473-s011.docx]

Multimedia Appendix 11. Confusion matrix of 3-category Cephalic Index -based head-shape classification between the 3D scanner and the mobile app at 42 months (n=216).

| 3D scanner＼App | Dolichocephaly | Normal | Brachycephaly | Row total |
| --- | --- | --- | --- | --- |
| Dolichocephaly | 5 (41.7) | 7 (58.3) | 0 (0.0) | 12 |
| Normal | 1 (0.5) | 171 (86.4) | 26 (13.1) | 198 |
| Brachycephaly | 0 (0.0) | 0 (0.0) | 6 (100.0) | 6 |
| Column total | 6 | 178 | 32 | 216 |

Values are n (row %).
