# Supplementary material for: Agreement and Screening Performance of an App-Based Cranial Shape Assessment Tool Compared With a 3D Scanner in Municipal Child Health Checkups in Japan: Instrument Validation Study
Source: JMIR Pediatr Parent. 2026 Oct 13;9:e105473. doi: 10.2196/105473 (PMC13662245; doi:10.2196/105473)
Supplement: Supplementary file 13 [file pediatrics-v9-e105473-s012.docx]

Multimedia Appendix 12. Direction of discordance between app- and 3D scanner–based 3-category Cephalic Index classifications (App < 3D scanner vs App > 3D scanner), by age group.

| Age group | Under-classification  (App < 3D scanner) | Exact agreement | Over-classification ^a^  (App > 3D scanner) | Total |
| --- | --- | --- | --- | --- |
| 4 months | 6 (2.3) | 176 (67.2) | 80 (30.5) | 262 |
| 18 months ^b^ | 0 (0.0) | 137 (80.6) | 33 (19.4) | 170 |
| 42 months | 1 (0.5) | 182 (84.3) | 33 (15.3) | 216 |

Values are n (valid %).

^a^ For Cephalic Index-based classification, overclassification indicates classification into a higher Cephalic Index category relative to the 3D scanner reference.

^b^ At 18 months, one case had missing Cephalic Index (total n=171; valid n=170).
