# Supplementary material for: Agreement and Screening Performance of an App-Based Cranial Shape Assessment Tool Compared With a 3D Scanner in Municipal Child Health Checkups in Japan: Instrument Validation Study
Source: JMIR Pediatr Parent. 2026 Oct 13;9:e105473. doi: 10.2196/105473 (PMC13662245; doi:10.2196/105473)
Supplement: Supplementary file 14 [file pediatrics-v9-e105473-s013.docx]

Multimedia Appendix 13. Device-stratified agreement between the 3D scanner and mobile app measurements of Cranial Vault Asymmetry Index (CVAI) and Cephalic Index at each assessment time point.

|  |  | iPad (May 22, 2023–Jun 24, 2024) | | | Lenovo (Jun 25, 2024–March 31, 2025) | | |
| --- | --- | --- | --- | --- | --- | --- | --- |
|  |  | 4 months (n=170) | 18 months (n=143^c^) | 42 months (n=108) | 4 months (n=92) | 18 months (n=28^d^) | 42 months (n=108) |
| CVAI | | | | | | | |
|  | Mean difference (app − scanner), mean (SD) | 0.17 (2.30) | 0.49 (3.13) | 0.38 (2.67) | 0.10 (2.02) | −0.55 (2.23) | 0.24 (2.38) |
|  | 95% limits of agreementª | −4.34 to 4.68 | −5.65 to 6.63 | −4.85 to 5.61 | −3.86 to 4.05 | −4.92 to 3.82 | −4.42 to 4.90 |
|  | ICC (95% CI)^b^ | 0.74  (0.67–0.80) | 0.41  (0.27–0.54) | 0.45  (0.28–0.59) | 0.81  (0.72–0.87) | 0.50  (0.17–0.73) | 0.54  (0.39–0.66) |
| Cephalic Index | | | | | | | |
|  | Mean difference (app − scanner), mean (SD) | 4.24 (3.45) | 3.51 (2.96) | 3.13 (3.04) | 3.06 (3.25) | 1.89 (3.27) | 2.10 (3.53) |
|  | 95% limits of agreementª | −2.52 to 11.00 | −2.29 to 9.31 | −2.83 to 9.09 | −3.30 to 9.43 | −4.52 to 8.30 | −4.82 to 9.02 |
|  | ICC (95% CI)^b^ | 0.63  (−0.02–0.85) | 0.68  (0.02–0.87) | 0.69  (0.11–0.87) | 0.81  (0.32–0.92) | 0.76  (0.46–0.89) | 0.70  (0.45–0.83) |

^a^ Limits of agreement were calculated as mean difference ± 1.96 × SD of the differences.
^b^ Intraclass correlation coefficients were calculated using a two-way mixed-effects model with absolute agreement; single measures.
^c^ At 18 months in the iPad period, one participant was excluded from the Cephalic Index analysis because of missing data (valid n=142 for CI), whereas CVAI analyses included n=143.
^d^ The Lenovo period included a small sample at 18 months (n=28); estimates for this subgroup should be interpreted with caution.
