# Supplementary material for: Agreement and Screening Performance of an App-Based Cranial Shape Assessment Tool Compared With a 3D Scanner in Municipal Child Health Checkups in Japan: Instrument Validation Study
Source: JMIR Pediatr Parent. 2026 Oct 13;9:e105473. doi: 10.2196/105473 (PMC13662245; doi:10.2196/105473)
Supplement: Supplementary file 15 [file pediatrics-v9-e105473-s014.docx]

Multimedia Appendix 14. Device-stratified sensitivity and specificity of the mobile app using the 3D scanner as the reference standard.

|  | 4 months | | | | 18 months | | | | 42 months | | | |
| --- | --- | --- | --- | --- | --- | --- | --- | --- | --- | --- | --- | --- |
|  | iPad Sens 95%CI ^a ,b^ | iPad Spec  95%CI | Lenovo Sens 95%CI | Lenovo Spec 95%CI | iPad Sens  95%CI | iPad Spec  95%CI | Lenovo Sens  95%CI^d^ | Lenovo Spec  95%CI | iPad Sens  95%CI | iPad Spec  95%CI | Lenovo Sens  95%CI | Lenovo Spec  95%CI |
| Plagiocephaly |  |  |  |  |  |  |  |  |  |  |  |  |
|  | 87.1  (27/31) 71.1–94.9 | 88.5  (123/139) 82.1–92.8 | 80.0  (12/15) 54.8–93.0 | 87.0  (67/77) 77.7–92.8 | 47.6  (10/21) 28.3–67.6 | 86.9  (106/122) 79.8–91.8 | 66.7  (2/3) 20.8–93.9 | 96.0  (24/25) 80.5–99.3 | 50.0  (3/6) 18.8–81.2 | 86.3  (88/102) 78.3–91.6 | 55.6  (5/9) 26.7–81.1 | 92.9  (92/99) 86.1–96.5 |
| Brachycephaly |  |  |  |  |  |  |  |  |  |  |  |  |
|  | 88.4  (38/43) 75.5–94.9 | 58.3  (74/127) 49.6–66.5 | 96.3  (26/27)  81.7–99.3 | 72.3  (47/65) 60.4–81.7 | 100.0  (9/9^c^) 70.1–100.0 | 87.2  (116/133) 80.5–91.9 | 100.0  (1/1) 20.7–100.0 | 81.5  (22/27) 63.3–91.8 | 100.0  (3/3) 43.9–100.0 | 85.7  (90/105) 77.8–91.1 | 100.0  (3/3) 43.9–100.0 | 89.5  (94/105) 82.2–94.0 |
| Dolichocephaly |  |  |  |  |  |  |  |  |  |  |  |  |
|  | 0.0  (0/5) 0.0–43.4 | 100.0  (165/165) 97.7–100.0 | 33.3  (2/6) 9.7–70.0 | 100.0  (86/86) 95.7–100.0 | 20.0  (2/10^c^) 5.7–51.0 | 100.0  (132/132) 97.2–100.0 | 0.0  (0/3) 0.0–56.1 | 100.0  (25/25) 86.7–100.0 | 33.3  (2/6) 9.7–70.0 | 100.0  (102/102) 96.4–100.0 | 50.0  (3/6) 18.8–81.2 | 99.0  (101/102) 94.7–99.8 |

^a^ Sensitivity and specificity are shown with the numerator/denominator in parentheses, followed by 95% confidence intervals.
^b^ 95% confidence intervals were calculated using the Wilson score method.
^c^ In the iPad period at 18 months, one participant was excluded from Cephalic Index-based analyses due to missing Cephalic Index (valid n=142); therefore, denominators for brachycephaly and dolichocephaly at 18 months reflect this reduced sample.
^d^ Estimates at 18 months in the Lenovo period (n=28) should be interpreted with caution due to the small sample size.

Note: Values in parentheses indicate the underlying numerators and denominators used to calculate each measure: TP/(TP+FN) for sensitivity and TN/(TN+FP) for specificity.
